# Positive Selection or Free to Vary? Assessing the Functional Significance of Sequence Change Using Molecular Dynamics

**DOI:** 10.1371/journal.pone.0147619

**Published:** 2016-02-12

**Authors:** Jane R. Allison, Marcus Lechner, Marc P. Hoeppner, Anthony M. Poole

**Affiliations:** 1 Centre for Theoretical Chemistry and Physics & Institute of Natural and Mathematical Sciences, Massey University Albany, Auckland, New Zealand; 2 Biomolecular Interaction Centre, University of Canterbury, Christchurch, New Zealand; 3 Maurice Wilkins Centre for Molecular Biodiscovery, Massey University Albany, Auckland, New Zealand; 4 Department of Pharmaceutical Chemistry, Philipps-University Marburg, Marburg, Germany; 5 Christian-Albrechts-University of Kiel, Institute of Clinical Molecular Biology, Kiel, Germany; 6 School of Biological Sciences, University of Canterbury, Christchurch, New Zealand; Indian Institute of Science, INDIA

## Abstract

Evolutionary arms races between pathogens and their hosts may be manifested as selection for rapid evolutionary change of key genes, and are sometimes detectable through sequence-level analyses. In the case of protein-coding genes, such analyses frequently predict that specific codons are under positive selection. However, detecting positive selection can be non-trivial, and false positive predictions are a common concern in such analyses. It is therefore helpful to place such predictions within a structural and functional context. Here, we focus on the p19 protein from tombusviruses. P19 is a homodimer that sequesters siRNAs, thereby preventing the host RNAi machinery from shutting down viral infection. Sequence analysis of the p19 gene is complicated by the fact that it is constrained at the sequence level by overprinting of a viral movement protein gene. Using homology modeling, in silico mutation and molecular dynamics simulations, we assess how non-synonymous changes to two residues involved in forming the dimer interface—one invariant, and one predicted to be under positive selection—impact molecular function. Interestingly, we find that both observed variation and potential variation (where a non-synonymous change to p19 would be synonymous for the overprinted movement protein) does not significantly impact protein structure or RNA binding. Consequently, while several methods identify residues at the dimer interface as being under positive selection, MD results suggest they are functionally indistinguishable from a site that is free to vary. Our analyses serve as a caveat to using sequence-level analyses in isolation to detect and assess positive selection, and emphasize the importance of also accounting for how non-synonymous changes impact structure and function.

## Introduction

Evolutionary arms races between host and pathogens can lead to rapid change in key genes associated with immune response or pathogenicity. In eukaryotes, RNA interference (RNAi) is a broadly conserved [1–6] mechanism that provides effective defense against a range of genomic pathogens, including viruses. Numerous viruses in turn encode viral suppressors of RNA silencing (VSR) that act via diverse mechanisms to suppress RNAi-mediated antiviral defense [7–9]. The distribution and variety of VSRs shows that viral suppressors have evolved numerous times independently, confirming the ongoing arms race between viruses and their hosts.

Evolutionary arms races may be detectable at the sequence level as positive selection. Positive selection is an excess of non-synonymous (amino-acid changing) nucleotide substitutions relative to synonymous substitutions (that do not affect protein sequence). Key genes involved in the RNAi response have been shown to be under positive selection [10], and it therefore seems reasonable to expect that VSR genes in viruses might also show evidence of positive selection.

Several factors complicate sequence-level analyses of positive selection. False positive predictions may arise when some synonymous sites are under greater constraint [11–13]. This may be a particular concern in viruses, where some protein-coding genes are known to be overprinted on other genes [11, 14, 15]. Overprinting refers to situations where two protein products, encoded in different frames or orientations, arise from the same nucleic acid sequence [15]. The de novo emergence of genes overprinted on existing genes is well documented in viruses [15–18]. Another complication, and the main focus of this study, is the impact that non-synonymous changes can have on function; sequence-level changes may not have equivalent effects on protein structure or function, and variation may be functionally significant even where a sequence is not under positive selection.

**Fig 1:**
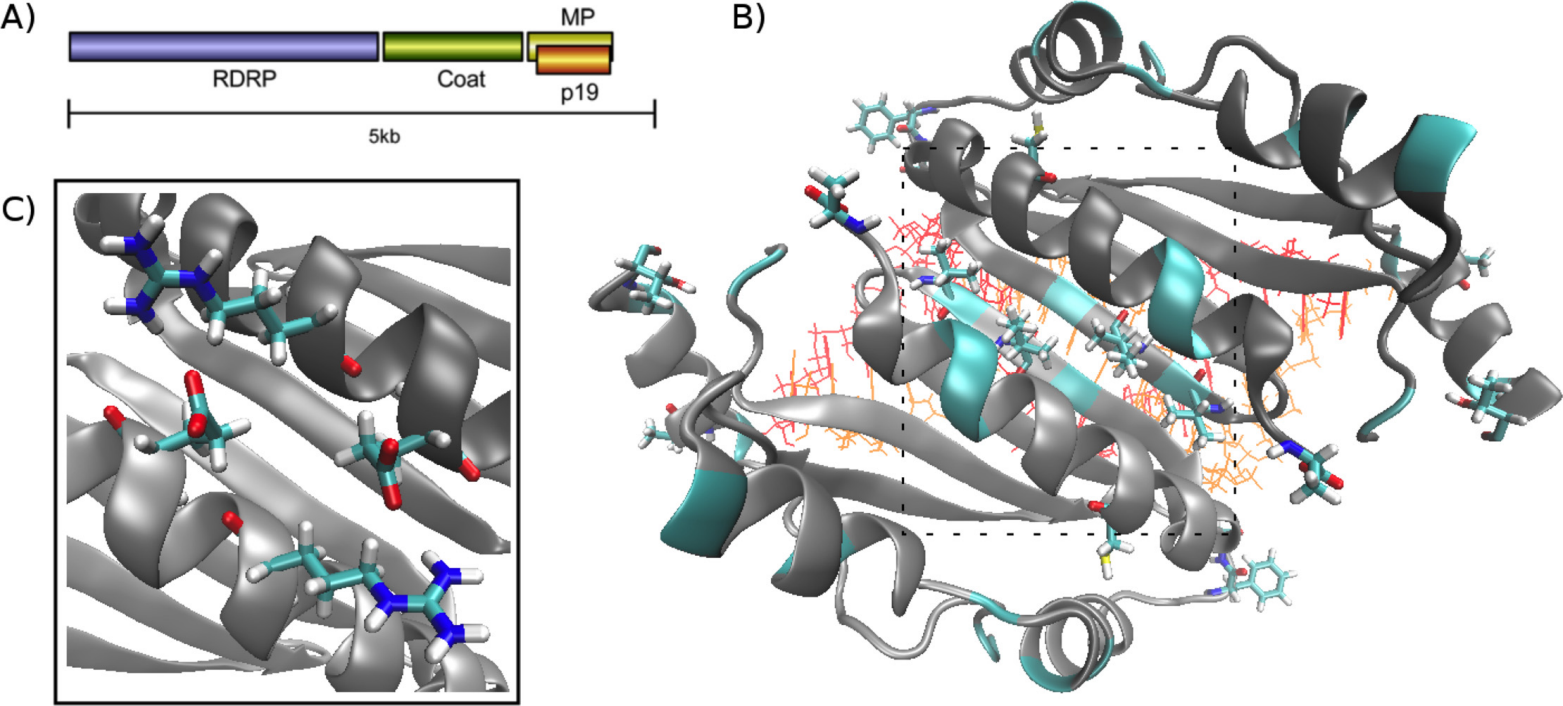
Tombusvirus genome structure and p19 crystal structure. (A) Schematic diagram showing the arrangement of the tombusvirus genome, including the overprinting of p19 on the movement protein (MP). (B) Crystal structure of the tomato bushy stunt (TBS) virus p19 dimer (PDB ID 1R9F) [24] bound to a 19 bp RNA fragment. The protein subunits are drawn in cartoon style and coloured dark and light grey; the two RNA strands are drawn as van der Waals spheres and coloured red and orange. Residues found to be under positive selection in at least two analyses are coloured cyan (cartoon representation), and residues identified as being under positive selection by all four analyses are drawn explicitly and coloured according to atom type (cyan: carbon; red: oxygen; blue: nitrogen; white: hydrogen). The dashed square indicates the region shown in panel C. (C) Close up view of the dimer interface with residues Arg139 and Glu143 of each subunit drawn explicitly.

Given concerns with false-positive predictions of positive selection [19–21], we have examined the intriguing case of p19, a viral suppressor of RNAi from the tombusvirus family of plant viruses [22]. P19 has significant constraints on sequence change due to the p19 gene being overprinted on the tombusvirus movement protein (MP) gene (**Fig 1A**). P19 is directly involved in viral suppression of the host RNAi machinery. It suppresses plant RNAi silencing by binding and sequestering siRNAs produced in response to viral infection [9, 22]. Structural studies indicate that p19 forms a homodimer that binds dsRNA in a sequence-independent but size-selective manner [23, 24], and this in turn prevents systemic spread of siRNA [25, 26].

P19 is therefore a clear candidate for participation in a host-pathogen arms race [7, 9]. Moreover, the p19 gene is overprinted on the MP gene in all known tombusvirus genomes [27] (**Fig 1A and Figure A in S1 File**). The structure of p19 is inherently linked to its function—it is an obligate dimer with respect to siRNA size selection and binding [23, 24]—and p19 appears to exhibit greater sequence diversity than MP. We therefore sought to establish whether p19 shows evidence of being subject to positive selection, as has been shown for other overprinted genes [14] and whether this signal appears genuine or could be an artifact of sequence level constraints attributable to its unusual genic organization. Following sequence level analyses to identify residues possibly under positive selection, we considered the structural and functional aspects of the identified sequence changes to give a more complete picture of their biological implications.

Sequence-based analyses yielded results consistent with p19 being under positive selection, despite the fact that MP overprinting constrains variation at p19 residues. Among sites putatively under positive selection, we chose to investigate those specifically involved in dimer formation. To establish how sequence changes impact function, we used molecular dynamics (MD) simulations to probe the effect on the p19 homodimer of all the observed natural variants at key interface residues (hereafter termed *observed*) plus any additional permissible mutations of these residues (where where nonsynonymous changes in p19 result in synonymous changes in the overprinted MP protein—hereafter *permissible*). Surprisingly, the majority of *observed* and *permissible* mutations of these sites do not impact dimer formation, even where the *permissible* mutations are expected to be disruptive, and have not been observed in nature. The robustness of the p19 dimeric structure to mutations suggests that while molecular evolutionary analyses support the inference of positive selection, the identification of positively selected sites provides only partial insight into structure and function. It may therefore be non-trivial to disentangle positive selection from sites that are simply robust to non-synonymous substitutions.

## Results and Discussion

### Sequence analysis suggests p19 may be under positive selection

**Fig 2:**
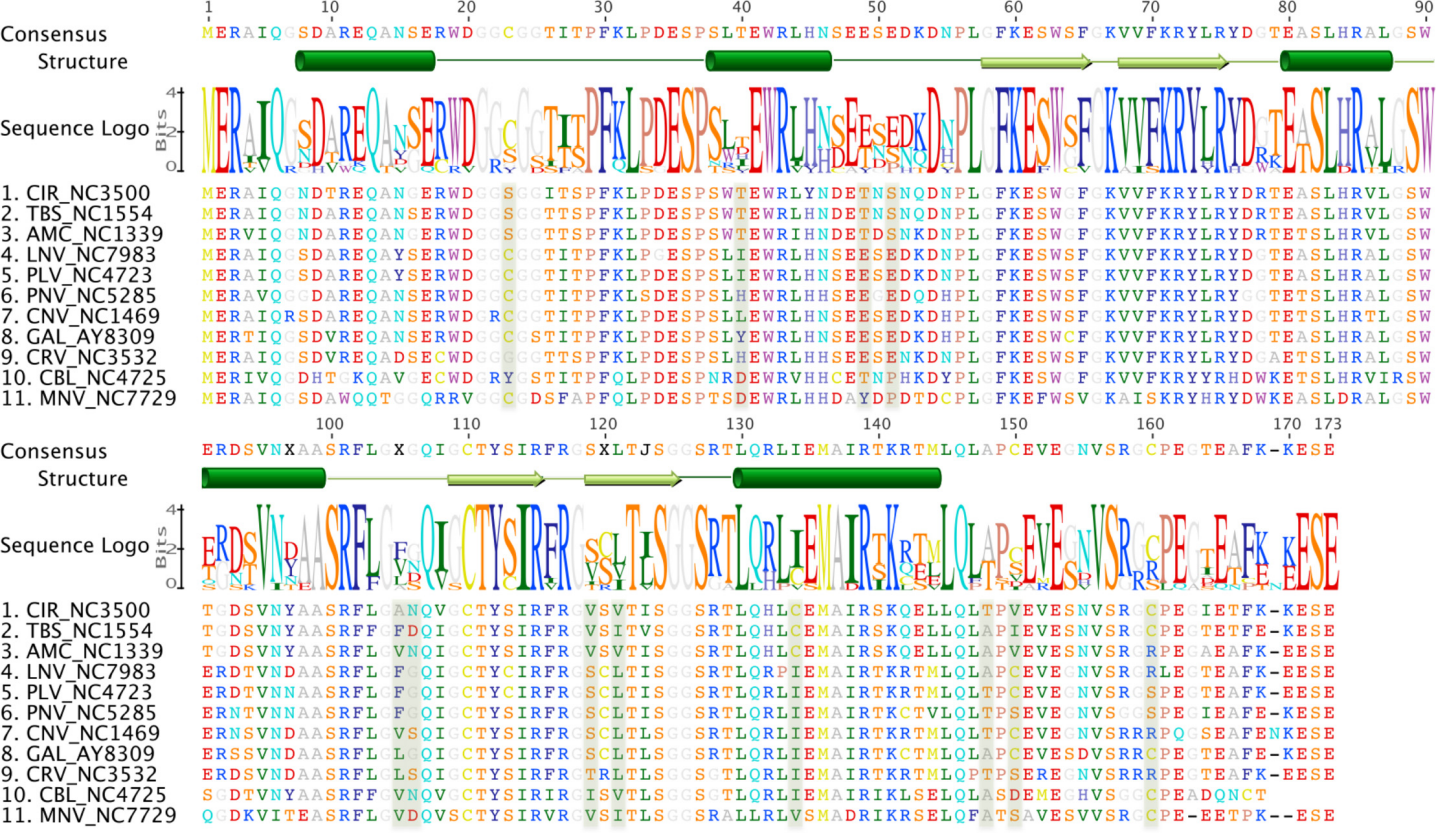
Alignment of amino acid sequences of p19 from 11 different tombusvirus species. The single-letter residue codes are coloured according to the nature of the amino acid side chain: (red) negatively-charged (D,E); (dark blue) aromatic (F,Y); (blue) positively-charged (K, R); (cyan) large polar amide-containing (Q,N); (orange) small polar hydroxyl-containing (S,T); (yellow) sulfur-containing (C,M); (grey) small aliphatic (A,G); (green) medium aliphatic (I,V,L); (purple-blue) imidazole (H); (violet) indole (W); (pink-brown) cyclised secondary amine (P). Sites identified as being under positive selection by all four analyses are highlighted in pale green. The secondary structure elements are indicated above the sequences: (barrels) α-helices; (arrows) β-strands. Viral species and NCBI accession numbers are as follows: CIR (Carnation italian ringspot virus, NC003500), TBS (Tomato bushy stunt virus, NC001554), AMC (Artichoke mottle crinkle virus, NC001339), LNV (Lisianthus necrosis virus, NC007983), PLV (Pear latent virus, NC004723), PNV (Pelagornium necrotic streak virus, NC005285), CNV (Cucumber necrosis virus, NC001469), GAL (Grapevine algerian latent virus, AY830918), CRV (Cymbidium ringspot virus, NC003532), CBL (Cucumber bulgarian latent virus, NC004725), MNV (Maize necrotic streak virus, NC007729).

**Fig 3:**
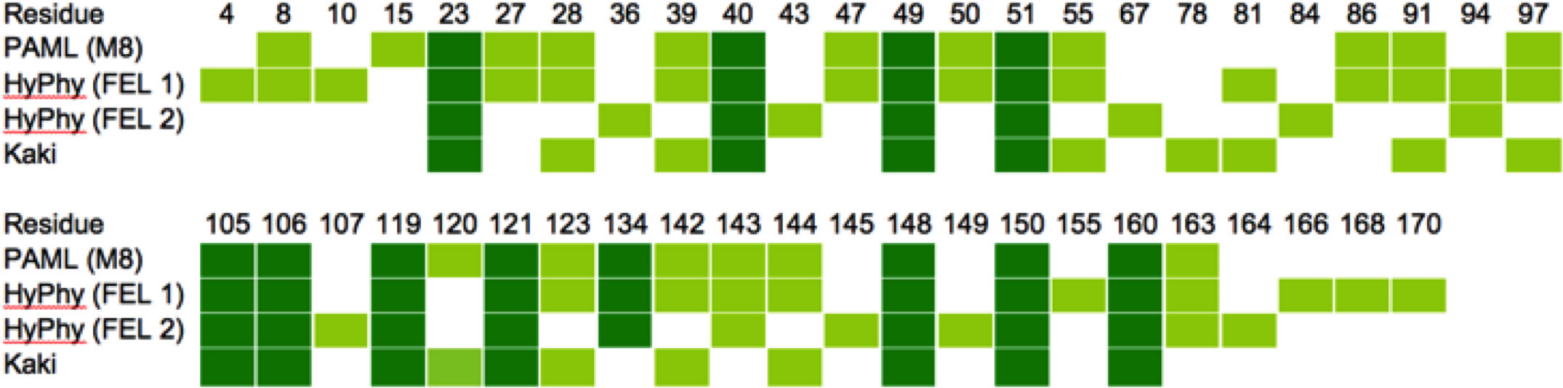
Residues identified as being under positive selection. See text for details of each of the four analyses. Light green indicates residues that only some of the analyses identified as being under positive selection. Dark green indicates residues found to be under positive selection in all four analyses. Note that some residues predicted to be under positive selection by HyPhy FEL2 (36, 43, 67, 107, 145) actually show no variation (see Fig 2).

To identify individual sites putatively under positive selection, we first analyzed available p19 sequences from tombusvirus whole genomes (**Fig 2 and Tables A-H and Figures A-C in S1 File**) using two Maximum Likelihood-based methods: Codeml, from the PAML package [28], and Fixed Effects Likelihood (FEL), implemented in the HyPhy package [20, 29, 30], a likelihood-based analogue to traditional, more conservative site-by-site counting methods. As p19 is overprinted, we also ran the Kaki package, which aims specifically to deal with more complex constraints on sequence evolution [12]. Notably, PAML and HyPhy both indicate that p19 is under positive selection, while Kaki results indicate that, when variable baseline substitution is taken into account, p19 is not under positive selection (**Tables I and J in S1 File**). That said, results from all three methods identified individual sites that are putatively under selection (**Fig 3**).

While there is a clear overlap in sites predicted to be under positive selection (**Fig 3**), each analysis yielded a number of potentially selected sites that were not picked up by one or more of the other methods. In contrast, PAML and HyPhy yielded no sites under positive selection in the MP or polymerase genes, which in fact appear to be under purifying selection (N.B. Kaki was not used here, as the polymerase gene is not overprinted). Our sequence-level analyses therefore suggest that p19 is under positive selection, whereas Kaki would predict this to be a false positive resulting from variable substitution rates. This latter result is expected given that p19 is overprinted on the MP gene.

### Location and functional relevance of residues under positive selection

As dN/dS has in some instances been shown to be a poor predictor of functionally important changes [31], we sought to further investigate the functional significance of residues under positive selection. We first examined their location in the tomato bushy stunt (TBS) virus p19 dimer (PDB ID 1R9F), for which a crystal structure has been solved [24] (**Fig 1B**). We found that a significant fraction of sites displaying positive selection are located in coil and loop regions or on the exterior of the protein. While this could suggest that ongoing residue substitutions at these sites serve to reduce detection of p19 by the host, it is not clear whether or how such host detection might occur. It is equally likely that the high variance observed for these residues is simply a result of their location in regions where a broad range of residue types are tolerated.

Only one of the residues identified as being under positive selection (**Fig 3**) is involved directly in siRNA binding. This residue (position 40) lies at the end of the caliper helix and forms hydrogen bonds to the terminal phosphate of the siRNA. Residue 39, a key component of the RNA “caliper”, was also found to be under positive selection in three of our analyses (**Fig 3**). However, the role of residue 39 is in siRNA size selection [23], and the natural variation at this site may in part be a reflection of the fact that multiple types of amino acids can perform this role [23].

While it is not known whether dimer formation occurs before, after, or concurrently with RNA binding, p19 is certainly an obligate dimer with respect to recognition of siRNAs of the correct length (~19–21 bp). Thus disruption or prevention of dimer formation represents a potential host defense mechanism, so the dimer interface is a likely location for positive selection to occur. Host defence might for example occur via a factor that directly blocks binding of the siRNA to the p19 dimer, or by binding at the dimer interface, thus preventing dimer formation and subsequent siRNA binding. This interface comprises the fourth β-strand (β4), which also lies at the center of the RNA-binding surface, and the fifth α-helix (α5) [24]. Both of these secondary structure elements contain several residues putatively under positive selection (**Fig 1B and Fig 3**).

Mutation of residues in β4 identified as being under positive selection (positions 119, 121 and 123) is unlikely to directly affect dimer formation, as the edge-to-edge linkage of β4 of each subunit to form the β-sheet that spans the dimer interface involves only the polypeptide backbone and is therefore sequence independent. However, highly disruptive mutations, such as to proline or amino acids with bulky or highly charged side chains, could have an indirect effect. Such changes are not observed in known tombusvirus sequences, however (**Figure C in S1 File**). Thus it is unclear whether positive selection of these dimer interface residues is symptomatic of an arms race in which the host is attempting to disrupt the interaction between β-strands across the dimer interface.

In contrast, the side chains of residues in α5 of each subunit interact across the dimer interface. Of particular note are residues 143 and 139, which form a pair of symmetrical salt bridges or multiple hydrogen bonds, depending on the nature of residue 143, across the dimer interface (**Fig 1C**). Interestingly, although residue 143 was identified as being under positive selection in three of our four analyses (**Fig 3**), residue 139 (Arg) is conserved in all known tombusvirus sequences (**Fig 2**). While two changes in Arg139 (to Trp or Gly) are *permissible* (**Figure C in S1 File**), these are expected to be highly disruptive. The *observed* variation in residue 143 is limited to amino acids that are capable of maintaining the contacts across the dimer interface (Ser/Thr/Glu). This is noteworthy given there is a wider range of *permissible* changes to residue 143 (**Figure C in S1 File**), some of which are predicted to disrupt the interaction with residue 139. This suggests a preference for amino acids capable of forming hydrogen bonds with the invariant Arg139.

From our sequence and structural analyses, it remained unclear whether selection restricts amino acid changes to some optimum subset of arrangements conducive to dimer formation, while negotiating other evolutionary pressures necessitated by an arms race and/or overprinting. We therefore created structures and carried out MD simulations of all *permissible* residue changes at sites 139 and 143, as well as all *observed* tombusvirus p19 sequences, with the aim of establishing whether the changes observed across residue 143 are indeed indicative of positive selection and whether, given that Arg139 is invariant, changes at this site impact p19 function.

### Evaluating the impact of dimer interface mutations

To test the effect of all *permissible* changes to residues 139 and 143, we created mutations within the context of the TBS tombusvirus sequence by directly mutating the crystal structure [24] in silico. Each sequence variant, including the wild-type TBS sequence, was simulated as a dimer both with and without a 19 bp siRNA bound. As a control, we simulated all *observed* tombusvirus p19 sequences (**Fig 2**) homology-modelled onto the TBS p19 crystal structure. This allowed us to test the assumption that other p19 sequences will form a structure similar to that of TBS p19. Importantly, it also allowed the effect of naturally occurring variants of residue 143 to be examined in context, where any potential disruption might be offset by compensatory mutations.

The homology modelling results confirm that the TBS p19 structure is likely to be shared by other known p19 proteins, with the most likely hit being the TBS p19 structure in all cases. Energy-minimisation and MD simulations of these *observed* variants showed them all to be structurally stable in the first instance, with no major loss of secondary, tertiary or quaternary structure in 50 ns of MD simulation with or without a siRNA bound (**Figures D-P in S1 File**). The caliper region shows the greatest flexibility (highest RMSF values), especially without the siRNA bound, as expected. Additionally, the structural stability of the dimer is nearly identical whether or not the siRNA is bound, other than the CBL, MNV and PLV proteins, where there was limited separation of the two subunits without RNA bound, resulting in large RMSD values, indicating that in most cases, RNA binding is not necessary for maintenance of p19 structure. Our assumption that other p19 sequences adopt a similar structure to that of TBS p19 is therefore valid.

To assess whether the *observed* variation in residue 143 requires buffering by compensatory mutations in order to maintain the overall structure, and therefore function, of p19, we examined the *permissible* single mutants of this residue and of the perfectly conserved residue Arg139 in the context of the TBS sequence (designated as wild-type hereafter). In this sequence, the combination of Glu143 and Arg139 maximises hydrogen-bonding capacity and allows for salt bridge formation.

**Fig 4:**
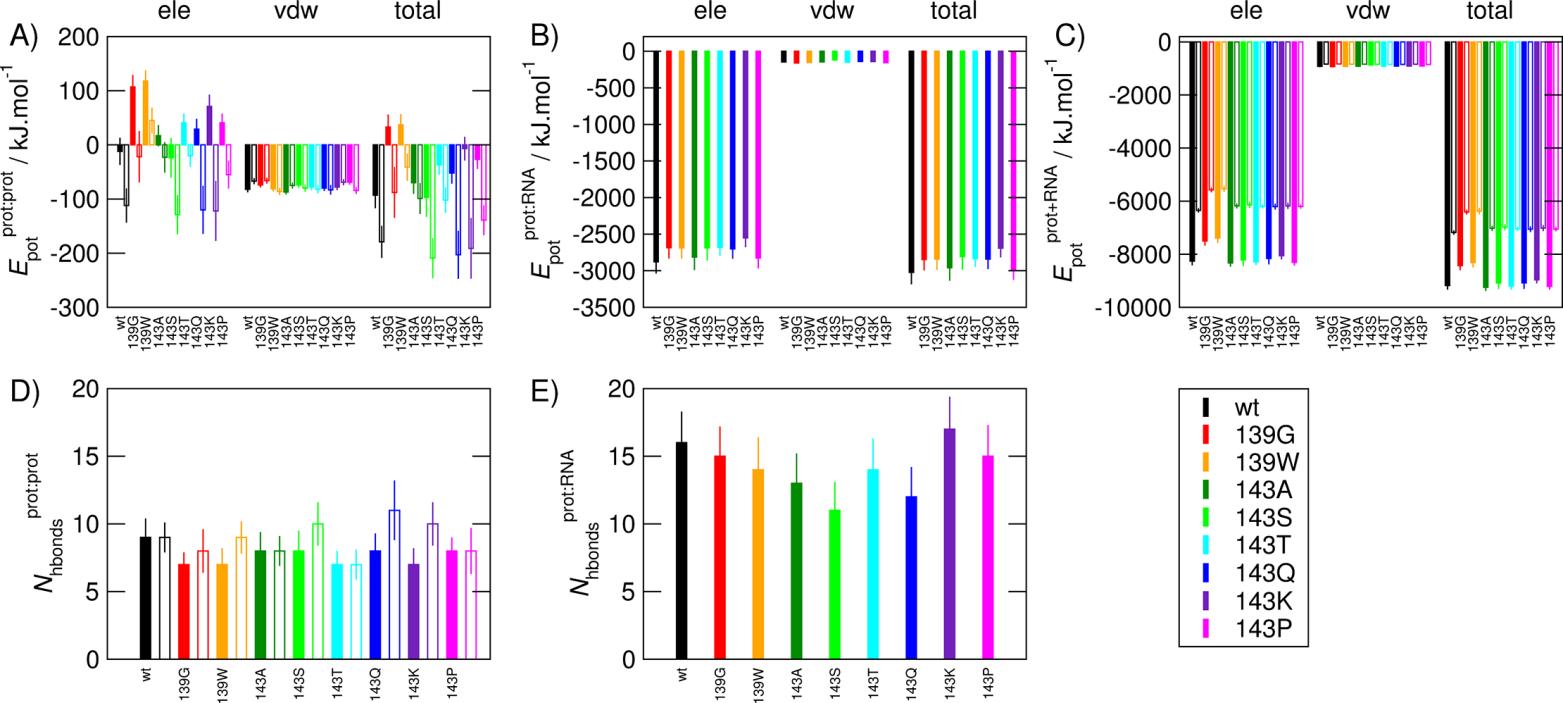
Potential energies and number of hydrogen bonds between different components of simulated p19 systems. Systems comprise wild-type and all *permissible* mutations of p19 alone or in complex with the siRNA. (A) Average potential energies (*E*_pot_) of the interaction between the two protein subunits making up the p19 dimer (prot:prot). (B) Average potential energies (*E*_pot_) of the interaction between the protein dimer and the RNA (prot;RNA). (C) Average potential energies (*E*_pot_) of the complete protein/RNA complex (prot+RNA). The electrostatic and van der Waals contributions are shown separately alongside the total potential energy, as indicated above the graphs. (D) Average number of hydrogen bonds during the entire simulation between the two protein subunits making up the p19 dimer. (E) Average number of hydrogen bonds during the entire simulation between the protein dimer and the RNA. Solid bars correspond to simulations with RNA included, and empty bars to simulations without RNA present. Averages were calculated from the first 50 ns of simulation, after 10 ns equilibration, so that the averages calculated from the simulations of p19 with RNA bound (200 ns) are comparable to those of the simulations without RNA bound (50 ns). The error bars correspond to the standard deviation in all cases.

We first assessed the effect of changes to the invariant residue Arg139 to determine whether MD simulations are capable of detecting undesirable substitutions, in this case the two *permissible* changes to this residue, both of which are expected to be highly disruptive. Although structurally stable, the Arg139 mutants lose the key salt bridge between residues 139 and 143 across the dimer interface (**Table K and Figure Z in S1 File**) and are the least energetically favourable (**Fig 4A–4C**), confirming that our simulations were sufficiently sensitive to detect the effects of unfavourable single amino acid changes. Additionally, the energetic effects of both *permissible* changes to Arg139 are much greater than those of even the most extreme changes to residue 143, suggesting that the complete conservation of Arg at position 139 is a result of selection rather than just chance.

Our sequence analyses suggested that residue 143 might be under positive selection (**Fig 3**), yet of the number of *permissible* substitutions at this position (Glu in the wild-type sequence), only three (Glu, Ser and Arg) are *observed*. Despite this, the overall structure of p19 was exceptionally resistant to changes to residue 143, with the structural properties of the mutants almost indistinguishable from those of the wild-type during the MD simulations (**Figures Q-Z in S1 File**). In almost all cases, the secondary, tertiary and quaternary structures were maintained throughout the simulations, with the caliper region exhibiting the greatest flexibility. One exception was the proline substitution, which, as expected, disrupted α5, providing further evidence that MD simulation is able to detect the effects of point mutations.

The attraction between the negatively charged RNA backbone and the positively-charged residues lining the RNA binding surface of the protein resulted in a large favourable protein-RNA interaction energy in all cases (**Fig 4B**). However, the protein-protein interaction energy (i.e. between subunits) of all *permissible* mutants is less favourable than that of the wild-type, and is in fact positive (i.e. unfavourable) when the siRNA is bound (**Fig 4A**). When the siRNA is not bound, the protein-protein interaction energy improves due to rearrangement of the dimer interface that increases the number and fractional occupancy of hydrogen bonds (**Fig 4D**) and, in some cases, salt bridges between monomers, whereas with the siRNA bound, only limited formation of alternative interactions is possible (**Table K and Figure Z in S1 File**). This is exemplified by the Glu143Ser mutant, which lost secondary structure both with and without the siRNA bound, but retained a protein-protein interaction energy similar to that of the wild-type due to forming a greater variety of hydrogen bonds (**Fig 4D, 4E and Figures V and Z and Table K in S1 File**). In comparison, in Glu143Gln, the similarity in the size and chemical nature of the side chain of the glutamine to that of the wild-type glutamate results in formation of a number of different interactions with residue 139 that mimic those formed by the wild-type Glu, but are more transient (**Table K and Figure Z in S1 File**). Thus while the *permissible* variants at position 143 are still able to maintain the dimer interface, for the more dramatic changes in the nature of residue 143, the structural rearrangements required to optimise the interactions spanning the interface are less feasible when the siRNA is bound. The extremely favourable electrostatic interactions associated with binding of the siRNA mean that the p19 dimer interface mutants are still functional, however.

Overall, our MD simulations showed p19 to be robust to changes to two key residues at the dimer interface, Arg139, which is highly conserved, and Glu143, suggested by sequence-level analyses and structural examination to be under positive selection. Ideally, the thermodynamic effect of the mutations should be quantified by calculation of binding free energies across the protein-protein interface. Such calculations are infeasible for systems of the size and number investigated here, but the interaction potential energies supported the structural analysis of the simulations. Together, these showed that what may seem to be positive selection at a sequence level and appear to be functionally relevant at a structural level is not necessarily functionally significant once protein dynamics are taken into account. In this particular case, rearrangement of the dimer interface is able to compensate for even highly disruptive mutations.

### Concluding Remarks

Detection of evolutionary arms races is of importance for understanding and managing host-pathogen relationships such as the competition between viruses and their plant or animal hosts. Arms races may be manifested as positive selection, which may be detected at a molecular level by the identification of fast-changing nucleotide sequences. Here, we sought to test whether sequence-level analyses are an appropriate means of detecting what is ultimately a phenotype-level effect. For this purpose, we chose a system where positive selection is expected to be occurring, namely the tombusvirus p19 protein, which is involved in suppression of the host plant RNAi response to viral infection. Importantly, the function of this protein depends largely on its structure, thus deconvoluting what is otherwise a complex relationship. Our sequence-level analyses did detect evidence of positive selection at a number of sites, and the structural context of these sites pointed to the dimer interface as being a key region in which positive selection resulting from an arms race might be taking place. We then evaluated the effect of mutations at the dimer interface by carrying out MD simulations of a comprehensive set of sequence variants. We observed a difference in the impact of mutations at a highly conserved residue compared to at a site putatively under positive selection, validating the use of MD to assess the effect of amino acid variation. The simulations of all *permissible* variations (given the constraints of overprinting) at a dimer interface site suggested to be under positive selection by sequence- and structural-level analyses revealed the p19 dimer to be robust to even highly disruptive changes to the dimer interface, calling into question the evolutionary impact of single mutations in this particular example. We therefore conclude that identifying and assessing the validity of positive selection can be greatly aided by taking protein structure and function into account. Generating mutants and assessing the functional impact on infection under containment would provide an independent means of establishing whether effects not detectable by MD simulations account for the lack of distinction we see between *observed* and *permissible* variants. However, as such experiments are often difficult to undertake, our MD simulations highlight the need for caution in interpreting positive selection detected at sequence level, and permit a more nuanced way of interpreting positive selection signals. In the specific case of p19, we conclude it is difficult to separate tolerance to variation from positive selection.

## Methods

### Phylogenetic analyses & tests of positive selection

Guide trees for the PAML and FEL analyses were constructed from tombusvirus polymerase sequences (RdRP) using Neighbor-Joining with p-distances (MEGA [32], 500 bootstrap replicates) and ML-based algorithms (PhyML [33], 500 bootstrap replicates) (**Figure A in S1 File**). A posterior probability of 95% under each guide tree was used as criterion for a site to be considered as being under positive selection. The optimal substitution model for the ML tree (GTR+G) was calculated with Modeltest [34]. For the codeml analysis, we compared NSsites models M1 and M7 to M2 and M8, respectively–as recommended by the authors [28]. A likelihood ratio test was conducted to decide which of the two respective models (neutral evolution vs. positive selection) fitted our data best (**Tables C-F in S1 File**). Both guide trees produced equivalent results in codeml with the exception of codon 64, which was hence excluded from further analyses. The FEL analysis was carried out assuming both a 1-rate (dS held constant) and 2-rate model (dS adjusted for each site), respectively [20, 29, 30]. Similar to the codeml analysis, sites were only considered as being under positive selection if they were retrieved by both guide trees. An equivalent analysis was performed for MP genes (**Tables A and B and Figure A in S1 File**). Kaki was run according to the authors’ instructions [12] as a means to address the complex constraints on the evolution of the overprinted p19 gene. Briefly, we tested for variability in the baseline substitution rate or whether substitution rate is homogeneous (M8-ρH vs M8-ρV and M8a-ρH vs M8a-ρV). We then tested for positive selection under both the assumption of sequence homogeneity (M8a-ρH vs M8-ρH) and where baseline substitution rate is variable (M8a-ρV vs M8-ρV) to assess whether there is selection at the DNA/RNA level. Results are presented in **Tables I and J in S1 File and Table M in S2 File**.

### Molecular dynamics simulations

The list of all *permissible* changes to residues Arg139 and Glu143 of the Tomato bushy stunt (TBS) tombusvirus p19 that do not change the amino acid sequence of the overprinted MP was created taking codon degeneracy into account (**Figure C in S1 File**). In all *permissible* sequence variants, two additional mutations, Leu144Met and Leu147Met, which were in the TBS tombusvirus p19 crystallised by Ye et al. [24] were also present. Initial coordinates for each new sequence were derived from this X-ray structure of p19 with a 19-bp siRNA fragment bound (1R9F) by in silico site-directed mutagenesis using VMD [35]. Initial coordinates for the *observed* tombusvirus p19 sequences were generated by homology modelling using the PHYRE2 Protein Fold Recognition Server in intensive modelling mode [36].

All simulations were carried out using the CHARMM27 all-atom force field [37, 38] and the NAMD software [39]. The lengths of all bonds involving hydrogen atoms were constrained using ShakeH [40] with a tolerance of 1.0 × 10^−9^ nm, allowing for an integration time step of 2 fs. Van der Waals interactions were smoothed to zero at a cut-off distance of 1.2 nm using a switching function initiated at 1.0 nm. Electrostatic interactions between pairs of atoms separated by one or two bonds were excluded, and those between atoms separated by three bonds were scaled. Long-range electrostatic interactions outside a cut-off distance of 1.2 nm were treated using particle mesh Ewald (PME) [41, 42] with a direct space tolerance of 10^−6^, interpolation order of 4 and grid spacing of 0.1 nm. The temperature was maintained at 293 K using the Langevin thermostat [43] with a damping coefficient γ of 1 ps^-1^. A constant pressure of 1.01325 bar was maintained using the Berendsen algorithm [44] with an isothermal compressibility of 2.755 × 10^−5^ (kJ mol^−1^ nm^−3^)^−1^, corresponding to a protein in water, and a relaxation time τ_p_ of 0.5 ps. Periodic boundary conditions were used.

The 1R9F X-ray structure and the initial structure predicted for each new sequence as detailed above, either with or without RNA bound, was energy-minimised in vacuum for 10,000 steps, then solvated in a cubic box of TIP3P water [45] with a minimum distance of 1.4 nm from the protein to the box edge using the VMD solvate package and neutralised by addition of sodium ions using the VMD autoionize package before a second 10,000 steps of energy-minimisation. The numbers of atoms protein, RNA, ion and solvent atoms and the simulation box dimensions are given in Table L in S1 File. The system was heated from 0 K to 293 K at a rate of 1 K every 2 ps (586 ps in total), followed by equilibration for 1.414 ns and finally a data collection phase of 50 ns (200 ns for all *permissible* mutations with RNA bound) during which coordinates were saved every 5 ps (100ps for 200ns simulations).

Analysis of the simulations was carried out using existing VMD packages (namdenergy, saltbr, hbonds) or analysis functions (rmsd, rmsf, sasa, secstruct) coupled to self-made tcl scripts (available upon request).

## Supporting Information

S1 File Supporting results for analyses presented in the main text. p19 dN/dS ratios (**Table A**). Movement Protein (MP) dN/dS ratios (**Table B**). PAML tests for positive selection, p19 (**Tables C and D**). PAML tests for positive selection, MP (**Tables E and F**). Codeml results (**Table G**). HyPhy results (**Table H**). Kaki results (**Tables I and J**). Formation of hydrogen bond and salt bridge interactions across the p19 dimer interface (**Table K**). Numbers of atoms and dimensions of simulation boxes (**Table L**). ML guide tree used for the PAML analysis, based on RDRP sequences from Tombusviruses (**Figure A**). dN/dS scatterplots (**Figure B**). Observed and permissible sequence variation (**Figure C**). Structural stability of all p19 variants studied with and without a 19 bp siRNA bound by molecular dynamics simulations (**Figures D-Y**). Potential energies and dimer interface stability of all *permissible* variants of the wild-type tomato bushy stunt virus p19 sequence with a 19 bp siRNA bound during 200 ns molecular dynamics simulation (**Figure Z**).
(DOC)

S2 File Results in excel format from analyses using Kaki. Raw results for all four models described in the text are presented **(Table M).**
(XLS)
