# Supplementary material for: Positive Selection or Free to Vary? Assessing the Functional Significance of Sequence Change Using Molecular Dynamics
Source: PLoS One. 2016 Feb 12;11(2):e0147619. doi: 10.1371/journal.pone.0147619 (PMC4752228; doi:10.1371/journal.pone.0147619)
Supplement: S1 File — p19 dN/dS ratios (Table A). Movement Protein (MP) dN/dS ratios (Table B). PAML tests for positive selection, p19 (Tables C and D). PAML tests for positive selection, MP (Tables E and F). Codeml results (Table G). HyPhy results (Table H). Kaki results (Tables I and J). Formation of hydrogen bond and salt bridge interactions across the p19 dimer interface (Table K). Numbers of atoms and dimensions of simulation boxes (Table L). ML guide tree used for the PAML analysis, based on RDRP sequences from Tombusviruses (Figure A). dN/dS scatterplots (Figure B). Observed and permissible sequence variation (Figure C). Structural stability of all p19 variants studied with and without a 19 bp siRNA bound by molecular dynamics simulations (Figures D-Y). Potential energies and dimer interface stability of all permissible variants of the wild-type tomato bushy stunt virus p19 sequence with a 19 bp siRNA bound during 200 ns molecular dynamics simulation (Figure Z). (DOC) [file pone.0147619.s001.doc]

Supporting information for:

**Positive selection or free to vary? Assessing the functional significance of sequence change using molecular dynamics.**

Jane R. Allison ^1,2,3^*, Marcus Lechner^4^, Marc P. Hoeppner^5^, Anthony M. Poole^2,6^*

^1^Centre for Theoretical Chemistry and Physics & Institute of Natural and Mathematical Sciences, Massey University Albany, Auckland, New Zealand

^2^Biomolecular Interaction Centre, University of Canterbury, Christchurch, New Zealand

^3^Maurice Wilkins Centre for Molecular Biodiscovery, Massey University Albany, Auckland, New Zealand

^4^Department of Pharmaceutical Chemistry, Philipps-University Marburg, Marburg, Germany

^5^Christian-Albrechts-University of Kiel, Institute of Clinical Molecular Biology, Kiel, Germany

^6^School of Biological Sciences, University of Canterbury, Christchurch, New Zealand

*Email: [j.allison@massey.ac.nz](mailto:j.allison@massey.ac.nz), [anthony.poole@canterbury.ac.nz](mailto:anthony.poole@canterbury.ac.nz)

### Table A: p19 dN/dS ratios (MEGA, NG) (p-distances)

| **AMC** |  |  |  |  |  |  |  |  |  |  |  |  |  |  |  |  |  |  |  |  |
| --- | --- | --- | --- | --- | --- | --- | --- | --- | --- | --- | --- | --- | --- | --- | --- | --- | --- | --- | --- | --- |
| **CBL** | 1.617* | *(0.16)* |  |  |  |  |  |  |  |  |  |  |  |  |  |  |  |  |  |  |
| **CIR** | 0.780 | *(0.04)* | 1.71* | (0.16) |  |  |  |  |  |  |  |  |  |  |  |  |  |  |  |  |
| **CNV** | 1.735* | *(0.13)* | 1.90* | (0.19) | 1.95* | (0.14) |  |  |  |  |  |  |  |  |  |  |  |  |  |  |
| **CRV** | 0.884 | *(0.15)* | 1.369 | (0.19) | 0.963 | (0.16) | 0.58 | (0.08) |  |  |  |  |  |  |  |  |  |  |  |  |
| **GAL** | 1.566 | *(0.14)* | 1.75* | (0.19) | 1.66* | (0.15) | 1.21 | (0.05) | 0.57 | (0.09) |  |  |  |  |  |  |  |  |  |  |
| **LNV** | 1.474 | *(0.13)* | 1.95* | (0.19) | 1.79* | (0.14) | 2.81* | (0.05) | 0.7 | (0.09) | 1.64 | (0.06) |  |  |  |  |  |  |  |  |
| **MNV** | 1.34* | *(0.22)* | 1.339 | (0.22) | 1.267 | (0.23) | 1.33 | (0.23) | 1.08 | (0.25) | 1.11 | (0.23) | 1.23 | (0.23) |  |  |  |  |  |  |
| **PLV** | 1.929* | *(0.12)* | 1.61* | (0.19) | 1.369 | (0.13) | 1.7 | (0.05) | 0.58 | (0.08) | 1.04 | (0.06) | 0.94 | (0.02) | 1.17 | (0.22) |  |  |  |  |
| **PNV** | 1.559* | *(0.15)* | 1.85* | (0.18) | 1.491 | (0.15) | 2.5* | (0.06) | 0.79 | (0.09) | 1.35 | (0.07) | 2.62* | (0.06) | 1.31 | (0.23) | 1.21 | (0.05) |  |  |
| **TBS** | 0.824 | *(0.04)* | 1.64* | (0.17) | 1.147 | (0.04) | 1.8* | (0.13) | 0.86 | (0.15) | 1.4 | (0.13) | 1.55* | (0.12) | 1.19 | (0.22) | 1.46 | (0.12) | 1.342 | (0.14) |

* under significant positive selection (p≤0.05), z-Test of selection (MEGA3)

### Table B: Movement Protein (MP) dN/dS ratios (MEGA, NG) (p-distances)

| **AMC** |  |  |  |  | |  | |  |  | |  |  |  |  |  |  |  |  |  |  |  |  |  |
| --- | --- | --- | --- | --- | --- | --- | --- | --- | --- | --- | --- | --- | --- | --- | --- | --- | --- | --- | --- | --- | --- | --- | --- |
| **CBL** | 0.208 | *(0.16)* |  | |  |  |  | |  |  | |  |  |  |  |  |  |  |  |  |  |  |  |
| **CIR** | 0.092 | *(0.04)* | 0.226 | | *(0.17)* |  |  | |  |  | |  |  |  |  |  |  |  |  |  |  |  |  |
| **CNV** | 0.224 | *(0.12)* | 0.194 | | *(0.19)* | 0.208 | *(0.13)* | |  |  | |  |  |  |  |  |  |  |  |  |  |  |  |
| **CRV** | 0.314 | *(0.15)* | 0.240 | | *(0.2)* | 0.274 | *(0.16)* | | 0.253 | *(0.08)* | |  |  |  |  |  |  |  |  |  |  |  |  |
| **GAL** | 0.223 | *(0.13)* | 0.183 | | *(0.19)* | 0.223 | *(0.14)* | | 0.097 | *(0.05)* | | 0.257 | *(0.09)* |  |  |  |  |  |  |  |  |  |  |
| **LNV** | 0.274 | *(0.12)* | 0.209 | | *(0.19)* | 0.251 | *(0.13)* | | 0.092 | *(0.05)* | | 0.313 | *(0.08)* | 0.178 | *(0.06)* |  |  |  |  |  |  |  |  |
| **MNV** | 0.345 | *(0.24)* | 0.266 | | *(0.24)* | 0.366 | *(0.24)* | | 0.271 | *(0.24)* | | 0.297 | *(0.26)* | 0.301 | *(0.24* | 0.310 | *(0.24)* |  |  |  |  |  |  |
| **PLV** | 0.249 | *(0.11)* | 0.196 | | *(0.19)* | 0.241 | *(0.13)* | | 0.044 | *(0.04)* | | 0.291 | *(0.08)* | 0.137 | *(0.06)* | 0.323 | *(0.02)* | 0.311 | *(0.23)* |  |  |  |  |
| **PNV** | 0.227 | *(0.14)* | 0.198 | | *(0.19)* | 0.223 | *(0.14)* | | 0.054 | *(0.05)* | | 0.203 | *(0.09)* | 0.099 | *(0.06)* | 0.096 | *(0.06)* | 0.293 | *(0.23)* | 0.075 | *(0.05)* |  |  |
| **TBSV** | 0.053 | *(0.04)* | 0.195 | | *(0.18)* | 0.088 | *(0.04)* | | 0.203 | *(0.13)* | | 0.275 | *(0.15)* | 0.247 | *(0.12)* | 0.268 | *(0.12)* | 0.365 | *(0.23)* | 0.258 | *(0.11)* | 0.229 | *(0.13)* |

### Table C: PAML test for positive selection, p19, NJ guide tree

| Site model | -lnL | p_d.f.=2_ |
| --- | --- | --- |
| M1a (nearly neutral) | -2538.689102 |  |
| M2a (positive selection) | -2452.544355 | <0.0001 |
| M7 (beta) | -2541.005722 |  |
| M8 (beta&ω) | -2452.551955 | <0.0001 |

### Table D: PAML test for positive selection, p19, ML guide tree

| Site model | -lnL | p_d.f.=2_ |
| --- | --- | --- |
| M1a (nearly neutral) | -2520.841452 |  |
| M2a (positive selection) | -2430.482980 | <0.0001 |
| M7 (beta) | -2523.050931 |  |
| M8 (beta&ω) | -2430.492645 | <0.0001 |

### Table E: PAML test for positive selection, MP, NJ guide tree

| Site model | -lnL | p_d.f.=2_ |
| --- | --- | --- |
| M1a (nearly neutral) | -2553.751295 |  |
| M2a (positive selection) | -2551.371829 | 0.09 |
| M7 (beta) | -2560.120251 |  |
| M8 (beta&ω) | -2552.117993 | 0.0002 |

### Table F: PAML test for positive selection, MP, ML guide tree

| Site model | -lnL | p_d.f.=2_ |
| --- | --- | --- |
| M1a (nearly neutral) | -2570.256157 |  |
| M2a (positive selection) | -2567.046702 | 0.09 |
| M7 (beta) | -2576.120343 |  |
| M8 (beta&ω) | -2567.503083 | 0.0002 |

### Table G: Codeml (M8, PAML) results^a^

| **Codeml** | | **ML guide tree** | |  |  | **NJ guide tree** | |  |  |
| --- | --- | --- | --- | --- | --- | --- | --- | --- | --- |
| **Codon** | **AA** | **Pr(w>1)** | **post mean** | **+** | **SE for w** | **Pr(w>1)** | **post mean** | **+** | **SE for w** |
| 4 | V | 0.873 | 6.696 | + | 2.332 | 0.907 | 6.703 | + | 1.984 |
| 8 | N | 0.995** | 7.546 | + | 0.899 | 0.996** | 7.302 | + | 0.808 |
| 10 | A | 0.836 | 6.442 | + | 2.56 | 0.884 | 6.55 | + | 2.16 |
| 15 | N | 0.962* | 7.316 | + | 1.483 | 0.978* | 7.177 | + | 1.18 |
| 23 | S | 0.999** | 7.575 | + | 0.793 | 0.999** | 7.319 | + | 0.741 |
| 27 | T | 0.967* | 7.35 | + | 1.416 | 0.942 | 6.939 | + | 1.651 |
| 28 | S | 0.988* | 7.496 | + | 1.06 | 0.958* | 7.048 | + | 1.465 |
| 39 | W | 1.000** | 7.581 | + | 0.765 | 1.000** | 7.325 | + | 0.715 |
| 40 | T | 1.000** | 7.581 | + | 0.765 | 1.000** | 7.325 | + | 0.715 |
| 44 | I | 0.546 | 4.464 | + | 3.372 | 0.526 | 4.181 | + | 3.245 |
| 47 | D | 1.000** | 7.581 | + | 0.766 | 1.000** | 7.325 | + | 0.715 |
| 49 | T | 1.000** | 7.581 | + | 0.765 | 1.000** | 7.325 | + | 0.715 |
| 50 | D | 0.999** | 7.574 | + | 0.796 | 0.999** | 7.319 | + | 0.737 |
| 51 | S | 1.000** | 7.581 | + | 0.766 | 1.000** | 7.325 | + | 0.715 |
| 52 | N | 0.935 | 7.128 | + | 1.802 | 0.897 | 6.633 | + | 2.066 |
| 53 | K | 0.606 | 4.871 | + | 3.291 | 0.848 | 6.31 | + | 2.404 |
| 55 | N | 0.974* | 7.401 | + | 1.305 | 0.978* | 7.18 | + | 1.174 |
| 64 | G | 0.748 | 5.833 | + | 2.946 | 0.964* | 7.084 | + | 1.386 |
| 78 | R | 0.829 | 6.394 | + | 2.594 | 0.881 | 6.524 | + | 2.185 |
| 81 | T | 0.916 | 6.997 | + | 1.993 | 0.948 | 6.979 | + | 1.587 |
| 86 | V | 0.977* | 7.423 | + | 1.253 | 0.984* | 7.216 | + | 1.081 |
| 91 | T | 1.000** | 7.581 | + | 0.765 | 1.000** | 7.325 | + | 0.715 |
| 92 | G | 0.861 | 6.612 | + | 2.411 | 0.892 | 6.601 | + | 2.102 |
| 93 | D | 0.513 | 4.22 | + | 3.305 | 0.666 | 5.097 | + | 3.051 |
| 94 | S | 0.661 | 5.244 | + | 3.205 | 0.949 | 6.987 | + | 1.577 |
| 97 | Y | 1.000** | 7.58 | + | 0.771 | 1.000** | 7.325 | + | 0.716 |
| 105 | V | 0.998** | 7.57 | + | 0.813 | 1.000** | 7.322 | + | 0.726 |
| 106 | N | 1.000** | 7.581 | + | 0.766 | 1.000** | 7.325 | + | 0.715 |
| 119 | V | 1.000** | 7.581 | + | 0.765 | 1.000** | 7.325 | + | 0.715 |
| 120 | S | 0.979* | 7.44 | + | 1.213 | 0.984* | 7.218 | + | 1.075 |
| 121 | V | 0.998** | 7.566 | + | 0.826 | 0.990* | 7.258 | + | 0.959 |
| 123 | I | 1.000** | 7.58 | + | 0.771 | 1.000** | 7.323 | + | 0.724 |
| 128 | R | 0.694 | 5.48 | + | 3.149 | 0.91 | 6.729 | + | 1.981 |
| 134 | C | 1.000** | 7.58 | + | 0.771 | 1.000** | 7.324 | + | 0.719 |
| 140 | S | 0.904 | 6.915 | + | 2.098 | 0.925 | 6.824 | + | 1.828 |
| 142 | Q | 1.000** | 7.581 | + | 0.766 | 1.000** | 7.325 | + | 0.716 |
| 143 | E | 1.000** | 7.581 | + | 0.768 | 1.000** | 7.324 | + | 0.717 |
| 144 | L | 0.983* | 7.467 | + | 1.142 | 0.997** | 7.307 | + | 0.788 |
| 148 | A | 0.977* | 7.419 | + | 1.262 | 0.955* | 7.024 | + | 1.506 |
| 150 | V | 1.000** | 7.581 | + | 0.765 | 1.000** | 7.325 | + | 0.715 |
|  |  |  |  |  |  | 0.545 | 4.303 | + | 3.209 |
|  |  |  |  |  |  | 0.9 | 6.651 | + | 2.043 |
| 155 | N | 0.693 | 5.445 | + | 3.107 | 0.777 | 5.829 | + | 2.729 |
|  |  |  |  |  |  | 0.555 | 4.367 | + | 3.203 |
| 160 | R | 0.989* | 7.503 | + | 1.04 | 0.996** | 7.295 | + | 0.832 |
| 163 | A | 1.000** | 7.579 | + | 0.774 | 1.000** | 7.323 | + | 0.724 |
| 165 | A | 0.947 | 7.216 | + | 1.674 | 0.917 | 6.767 | + | 1.905 |
| 167 | K | 0.935 | 7.132 | + | 1.804 | 0.972* | 7.138 | + | 1.279 |

^a^Yellow highlighting indicates cases where Pr(w>1)≥0.95

### Table H: FEL results (1-rate and 2-rates, HyPhy)^a^

| **FEL** | **1 ratio, ML** | |  |  | **1 ratio, NJ** |  |  |  | **2 ratio, ML** | |  |  | **2 ratio, NJ** | |  |
| --- | --- | --- | --- | --- | --- | --- | --- | --- | --- | --- | --- | --- | --- | --- | --- |
| **Codon** | **dN/dS** | **LRT** | **p-value** | **Log(L)** | **dN/dS** | **LRT** | **p-value** | **Log(L)** | **LRT** | **p-value** | **Full Log(L)** | **dS=dN** | **LRT** | **p-value** | **Full Log(L)** |
| 1 | 1.000 | 0.0000 | 1.000 | 0 | 1.000 | 0.0000 | 1.000 | 0 | 0.0000 | 1.000 | 0 |  | 0.0000 | 1.000 | 0 |
| 2 | 0.000 | 3.0287 | 0.041 | -13.504 | 0.000 | 2.4368 | 0.059 | -15.2649 | ##### | 0.000 | -9.47025 |  | 11.0634 | 0.001 | -9.47025 |
| 3 | 1.000 | 0.0000 | 1.000 | 0 | 1.000 | 0.0000 | 1.000 | 0 | 0.0000 | 1.000 | 0 |  | 0.0000 | 1.000 | 0 |
| 4 | 3.804 | 4.5448 | 0.017 | -16.761 | 5.159 | 6.2507 | 0.006 | -17.5084 | 3.2643 | 0.071 | -16.6376 |  | 3.2969 | 0.069 | -16.1984 |
| 5 | 1.346 | 0.1532 | 0.348 | -12.218 | 1.684 | 0.4360 | 0.255 | -12.1771 | 0.8528 | 0.356 | -10.9068 |  | 0.8211 | 0.365 | -11.8776 |
| 6 | 1.000 | 0.0000 | 1.000 | 0 | 1.000 | 0.0000 | 1.000 | 0 | 0.0000 | 1.000 | 0 |  | 0.0000 | 1.000 | 0 |
| 7 | 0.838 | 0.0316 | 0.429 | -9.6761 | 1.028 | 0.0007 | 0.489 | -9.71316 | 0.8049 | 0.370 | -9.15714 |  | 0.8033 | 0.370 | -9.08739 |
| 8 | 5.107 | 8.8357 | 0.001 | -26.156 | 3.853 | 4.4241 | 0.018 | -21.9945 | 0.0205 | 0.886 | -23.5537 |  | 0.0132 | 0.909 | -24.7214 |
| 9 | 0.664 | 0.1858 | 0.333 | -10.12 | 0.842 | 0.0299 | 0.431 | -10.0039 | 0.5649 | 0.452 | -9.65402 |  | 0.5634 | 0.453 | -9.63155 |
| 10 | 3.608 | 4.2535 | 0.020 | -17.405 | 4.921 | 5.9035 | 0.008 | -18.2357 | 3.2455 | 0.072 | -17.3344 |  | 3.1913 | 0.074 | -16.8356 |
| 11 | 1.407 | 0.2013 | 0.327 | -12.017 | 1.830 | 0.5824 | 0.223 | -11.5967 | 1.1741 | 0.279 | -11.5536 |  | 1.1724 | 0.279 | -11.5299 |
| 12 | 1.396 | 0.1921 | 0.331 | -11.323 | 1.814 | 0.5636 | 0.226 | -10.9723 | 0.7137 | 0.398 | -11.0639 |  | 0.7101 | 0.399 | -11.0423 |
| 13 | 1.000 | 0.0000 | 1.000 | 0 | 1.000 | 0.0000 | 1.000 | 0 | 0.0000 | 1.000 | 0 |  | 0.0000 | 1.000 | 0 |
| 14 | 0.794 | 0.0561 | 0.406 | -8.2133 | 0.983 | 0.0004 | 0.492 | -7.64893 | 0.7481 | 0.387 | -7.61988 |  | 0.7497 | 0.387 | -7.64339 |
| 15 | 4.286 | 6.2212 | 0.006 | -27.153 | 5.878 | 8.6788 | 0.002 | -27.6021 | 0.1243 | 0.724 | -26.6986 |  | 0.1134 | 0.736 | -25.5771 |
| 16 | 2.217 | 1.4247 | 0.116 | -13.153 | 1.915 | 0.6498 | 0.210 | -12.4063 | 1.5659 | 0.211 | -15.5157 |  | 1.5499 | 0.213 | -12.7439 |
| 17 | 0.692 | 0.1488 | 0.350 | -9.0849 | 0.882 | 0.0158 | 0.450 | -8.74612 | 0.3589 | 0.549 | -8.78465 |  | 0.3578 | 0.550 | -8.80375 |
| 18 | 1.646 | 0.4081 | 0.261 | -12.985 | 2.145 | 0.8825 | 0.174 | -12.4908 | 1.4713 | 0.225 | -12.5881 |  | 1.4579 | 0.227 | -12.4451 |
| 19 | 0.842 | 0.0307 | 0.430 | -8.7825 | 1.107 | 0.0098 | 0.461 | -8.28485 | 0.1216 | 0.727 | -8.68345 |  | 0.1193 | 0.730 | -8.71016 |
| 20 | 0.679 | 0.1641 | 0.343 | -11.885 | 0.881 | 0.0162 | 0.449 | -11.3148 | 1.2252 | 0.268 | -11.2937 |  | 1.2388 | 0.266 | -11.3292 |
| 21 | 1.000 | 0.0000 | 1.000 | 0 | 1.000 | 0.0000 | 1.000 | 0 | 0.0000 | 1.000 | 0 |  | 0.0000 | 1.000 | 0 |
| 22 | 1.825 | 0.5727 | 0.225 | -12.378 | 2.265 | 0.9839 | 0.161 | -12.2148 | 1.6675 | 0.197 | -11.8818 |  | 1.6550 | 0.198 | -11.8092 |
| 23 | 25.894 | 15.1780 | 0.000 | -43.823 | 5.655 | 4.3952 | 0.018 | -33.6435 | ##### | 0.000 | -30.6696 |  | 31.7467 | 0.000 | -30.5345 |
| 24 | 1.000 | 0.0000 | 1.000 | 0 | 1.000 | 0.0000 | 1.000 | 0 | 0.0000 | 1.000 | 0 |  | 0.0000 | 1.000 | 0 |
| 25 | 2.574 | 1.9173 | 0.083 | -13.689 | 3.160 | 2.6692 | 0.051 | -12.94 | 2.0657 | 0.151 | -12.184 |  | 2.1377 | 0.144 | -13.178 |
| 26 | 1.593 | 0.3585 | 0.275 | -16.972 | 2.052 | 0.7935 | 0.187 | -18.1084 | 0.0173 | 0.895 | -18.4978 |  | 0.0159 | 0.900 | -16.9295 |
| 27 | 4.131 | 5.8160 | 0.008 | -20.476 | 3.278 | 2.7817 | 0.048 | -15.4215 | 2.9384 | 0.086 | -18.9453 |  | 3.0903 | 0.079 | -20.0041 |
| 28 | 4.437 | 5.7181 | 0.008 | -22.221 | 3.310 | 2.8354 | 0.046 | -15.6603 | 3.5514 | 0.059 | -19.6079 |  | 3.7852 | 0.052 | -21.6533 |
| 29 | 1.000 | 0.0000 | 1.000 | 0 | 1.000 | 0.0000 | 1.000 | 0 | 0.0000 | 1.000 | 0 |  | 0.0000 | 1.000 | 0 |
| 30 | 1.000 | 0.0000 | 1.000 | 0 | 1.000 | 0.0000 | 1.000 | 0 | 0.0000 | 1.000 | 0 |  | 0.0000 | 1.000 | 0 |
| 31 | 0.681 | 0.1641 | 0.343 | -9.7138 | 0.897 | 0.0119 | 0.457 | -10.036 | 0.3537 | 0.552 | -9.49904 |  | 0.3524 | 0.553 | -9.43294 |
| 32 | 0.000 | 2.5031 | 0.057 | -8.3029 | 0.000 | 1.9409 | 0.082 | -8.13023 | 2.4709 | 0.116 | -8.10113 |  | 2.4718 | 0.116 | -8.12017 |
| 33 | 0.777 | 0.0681 | 0.397 | -14.179 | 0.994 | 0.0000 | 0.497 | -14.404 | 0.3565 | 0.550 | -13.199 |  | 0.3456 | 0.557 | -14.0337 |
| 34 | 0.632 | 0.2387 | 0.313 | -11.227 | 0.798 | 0.0530 | 0.409 | -10.6886 | 0.5439 | 0.461 | -10.7238 |  | 0.5418 | 0.462 | -10.7366 |
| 35 | 1.000 | 0.0000 | 1.000 | 0 | 1.000 | 0.0000 | 1.000 | 0 | 0.0000 | 1.000 | 0 |  | 0.0000 | 1.000 | 0 |
| 36 | 0.000 | 3.3475 | 0.034 | -7.2741 | 0.000 | 2.6845 | 0.051 | -7.61343 | 3.9252 | 0.048 | -6.75598 |  | 3.8883 | 0.049 | -6.71656 |
| 37 | 1.000 | 0.0000 | 1.000 | 0 | 1.000 | 0.0000 | 1.000 | 0 | 0.0000 | 1.000 | 0 |  | 0.0000 | 1.000 | 0 |
| 38 | 1.305 | 0.1251 | 0.362 | -11.498 | 1.700 | 0.4578 | 0.249 | -11.1384 | 0.7579 | 0.384 | -11.2008 |  | 0.7549 | 0.385 | -11.1781 |
| 39 | 17.519 | 36.9962 | 0.000 | -35.219 | 6.853 | 8.0210 | 0.002 | -22.4516 | 1.5672 | 0.211 | -34.1989 |  | 3.6829 | 0.055 | -35.0092 |
| 40 | 24.942 | 31.9176 | 0.000 | -32.709 | 18.613 | 21.3831 | 0.000 | -28.9365 | 8.6066 | 0.003 | -28.6374 |  | 10.1852 | 0.001 | -32.2851 |
| 41 | 1.000 | 0.0000 | 1.000 | 0 | 1.000 | 0.0000 | 1.000 | 0 | 0.0000 | 1.000 | 0 |  | 0.0000 | 1.000 | 0 |
| 42 | 1.000 | 0.0000 | 1.000 | 0 | 1.000 | 0.0000 | 1.000 | 0 | 0.0000 | 1.000 | 0 |  | 0.0000 | 1.000 | 0 |
| 43 | 0.000 | 2.6969 | 0.050 | -17.21 | 0.000 | 2.2498 | 0.067 | -15.1046 | 8.2703 | 0.004 | -17.3779 |  | 7.3879 | 0.007 | -14.5864 |
| 44 | 2.686 | 1.4133 | 0.117 | -19.63 | 3.806 | 2.3024 | 0.065 | -21.4891 | 0.0013 | 0.972 | -19.6312 |  | 0.0125 | 0.911 | -19.1312 |
| 45 | 0.641 | 0.2256 | 0.317 | -9.0922 | 0.813 | 0.0445 | 0.416 | -10.5297 | 0.3429 | 0.558 | -10.2501 |  | 0.3443 | 0.557 | -8.80166 |
| 46 | 2.086 | 1.2157 | 0.135 | -19.554 | 2.772 | 2.1212 | 0.073 | -19.8884 | 1.7591 | 0.185 | -18.0599 |  | 1.7211 | 0.190 | -19.0666 |
| 47 | 7.623 | 19.1933 | 0.000 | -21.941 | 4.344 | 5.3305 | 0.010 | -15.3686 | 3.8509 | 0.050 | -21.2141 |  | 3.7983 | 0.051 | -21.6087 |
| 48 | 0.707 | 0.1305 | 0.359 | -9.6382 | 0.911 | 0.0086 | 0.463 | -9.27211 | 0.5538 | 0.457 | -9.17129 |  | 0.5514 | 0.458 | -9.19095 |
| 49 | 11.665 | 27.7298 | 0.000 | -28.373 | 6.546 | 8.5852 | 0.002 | -19.9466 | 6.7652 | 0.009 | -25.9415 |  | 6.1499 | 0.013 | -28.0024 |
| 50 | 5.503 | 11.4118 | 0.000 | -20.392 | 3.678 | 4.2315 | 0.020 | -16.9997 | 2.7305 | 0.098 | -20.1549 |  | 2.7316 | 0.098 | -20.0812 |
| 51 | 12.324 | 22.5064 | 0.000 | -30.749 | 3.494 | 3.0352 | 0.041 | -16.9093 | ##### | 0.002 | -26.8706 |  | 9.0618 | 0.003 | -30.1587 |
| 52 | 3.768 | 5.1512 | 0.012 | -19.146 | 2.777 | 2.1558 | 0.071 | -14.1756 | 1.7180 | 0.190 | -17.6743 |  | 1.8400 | 0.175 | -18.855 |
| 53 | 2.932 | 2.3245 | 0.064 | -27.163 | 3.181 | 2.5132 | 0.056 | -26.8355 | 1.0750 | 0.300 | -25.327 |  | 1.2106 | 0.271 | -24.909 |
| 54 | 1.000 | 0.0000 | 1.000 | 0 | 1.000 | 0.0000 | 1.000 | 0 | 0.0000 | 1.000 | 0 |  | 0.0000 | 1.000 | 0 |
| 55 | 4.187 | 6.0911 | 0.007 | -25.009 | 4.723 | 5.8180 | 0.008 | -20.8518 | 2.0472 | 0.152 | -22.6513 |  | 2.0096 | 0.156 | -24.7197 |
| 56 | 1.000 | 0.0000 | 1.000 | 0 | 1.000 | 0.0000 | 1.000 | 0 | 0.0000 | 1.000 | 0 |  | 0.0000 | 1.000 | 0 |
| 57 | 1.000 | 0.0000 | 1.000 | 0 | 1.000 | 0.0000 | 1.000 | 0 | 0.0000 | 1.000 | 0 |  | 0.0000 | 1.000 | 0 |
| 58 | 1.000 | 0.0000 | 1.000 | 0 | 1.000 | 0.0000 | 1.000 | 0 | 0.0000 | 1.000 | 0 |  | 0.0000 | 1.000 | 0 |
| 59 | 1.000 | 0.0000 | 1.000 | 0 | 1.000 | 0.0000 | 1.000 | 0 | 0.0000 | 1.000 | 0 |  | 0.0000 | 1.000 | 0 |
| 60 | 1.000 | 0.0000 | 1.000 | 0 | 1.000 | 0.0000 | 1.000 | 0 | 0.0000 | 1.000 | 0 |  | 0.0000 | 1.000 | 0 |
| 61 | 1.000 | 0.0000 | 1.000 | 0 | 1.000 | 0.0000 | 1.000 | 0 | 0.0000 | 1.000 | 0 |  | 0.0000 | 1.000 | 0 |
| 62 | 1.378 | 0.1765 | 0.337 | -13.819 | 1.814 | 0.5634 | 0.226 | -13.0868 | 1.1706 | 0.279 | -13.2835 |  | 1.1659 | 0.280 | -13.324 |
| 63 | 1.000 | 0.0000 | 1.000 | 0 | 1.000 | 0.0000 | 1.000 | 0 | 0.0000 | 1.000 | 0 |  | 0.0000 | 1.000 | 0 |
| 64 | 3.806 | 5.1292 | 0.012 | -18.924 | 2.800 | 2.1896 | 0.069 | -14.7488 | 2.0942 | 0.148 | -16.0295 |  | 2.3741 | 0.123 | -18.5429 |
| 65 | 0.672 | 0.1779 | 0.337 | -10.134 | 0.895 | 0.0126 | 0.455 | -9.72501 | 0.5979 | 0.439 | -9.6008 |  | 0.5948 | 0.441 | -9.62185 |
| 66 | 1.000 | 0.0000 | 1.000 | 0 | 1.000 | 0.0000 | 1.000 | 0 | 0.0000 | 1.000 | 0 |  | 0.0000 | 1.000 | 0 |
| 67 | 0.000 | 3.1972 | 0.037 | -6.9956 | 0.000 | 2.5714 | 0.054 | -6.73769 | 4.0047 | 0.045 | -6.33537 |  | 4.0184 | 0.045 | -6.35243 |
| 68 | 0.810 | 0.0460 | 0.415 | -8.4719 | 1.010 | 0.0001 | 0.496 | -7.89864 | 0.7612 | 0.383 | -7.8774 |  | 0.7620 | 0.383 | -7.90211 |
| 69 | 0.815 | 0.0428 | 0.418 | -11.621 | 1.017 | 0.0003 | 0.493 | -10.9838 | 0.3659 | 0.545 | -11.3988 |  | 0.3635 | 0.547 | -11.4425 |
| 70 | 0.654 | 0.2039 | 0.326 | -9.0722 | 0.848 | 0.0284 | 0.433 | -8.53846 | 0.3795 | 0.538 | -8.73166 |  | 0.3781 | 0.539 | -8.75788 |
| 71 | 1.000 | 0.0000 | 1.000 | 0 | 1.000 | 0.0000 | 1.000 | 0 | 0.0000 | 1.000 | 0 |  | 0.0000 | 1.000 | 0 |
| 72 | 1.000 | 0.0000 | 1.000 | 0 | 1.000 | 0.0000 | 1.000 | 0 | 0.0000 | 1.000 | 0 |  | 0.0000 | 1.000 | 0 |
| 73 | 1.000 | 0.0000 | 1.000 | 0 | 1.000 | 0.0000 | 1.000 | 0 | 0.0000 | 1.000 | 0 |  | 0.0000 | 1.000 | 0 |
| 74 | 1.711 | 0.4605 | 0.249 | -13.864 | 2.338 | 1.0197 | 0.156 | -13.807 | 1.8133 | 0.178 | -13.2643 |  | 1.8077 | 0.179 | -13.1854 |
| 75 | 1.000 | 0.0000 | 1.000 | 0 | 1.000 | 0.0000 | 1.000 | 0 | 0.0000 | 1.000 | 0 |  | 0.0000 | 1.000 | 0 |
| 76 | 0.770 | 0.0730 | 0.394 | -9.6906 | 0.969 | 0.0010 | 0.487 | -9.40242 | 0.6422 | 0.423 | -9.21767 |  | 0.6403 | 0.424 | -9.20259 |
| 77 | 0.635 | 0.2337 | 0.314 | -9.5134 | 0.800 | 0.0519 | 0.410 | -9.68821 | 0.5690 | 0.451 | -8.04305 |  | 0.5503 | 0.458 | -9.0178 |
| 78 | 3.427 | 3.9781 | 0.023 | -17.135 | 2.089 | 0.8333 | 0.181 | -12.4958 | 2.4163 | 0.120 | -16.1488 |  | 2.5546 | 0.110 | -16.6789 |
| 79 | 1.549 | 0.3084 | 0.289 | -20.049 | 2.087 | 0.7715 | 0.190 | -20.4496 | 0.9112 | 0.340 | -19.0541 |  | 0.8989 | 0.343 | -18.7637 |
| 80 | 1.000 | 0.0000 | 1.000 | 0 | 1.000 | 0.0000 | 1.000 | 0 | 0.0000 | 1.000 | 0 |  | 0.0000 | 1.000 | 0 |
| 81 | 4.158 | 5.5292 | 0.009 | -18.497 | 4.713 | 5.6997 | 0.008 | -18.066 | 3.6931 | 0.055 | -18.4012 |  | 3.6260 | 0.057 | -17.9277 |
| 82 | 1.000 | 0.0000 | 1.000 | 0 | 1.000 | 0.0000 | 1.000 | 0 | 0.0000 | 1.000 | 0 |  | 0.0000 | 1.000 | 0 |
| 83 | 0.000 | 2.1094 | 0.073 | -19.673 | 0.000 | 1.6309 | 0.101 | -17.7446 | 8.7638 | 0.003 | -18.3367 |  | 7.8285 | 0.005 | -15.7108 |
| 84 | 0.659 | 0.1956 | 0.329 | -9.6947 | 0.859 | 0.0235 | 0.439 | -9.30608 | 0.5628 | 0.453 | -9.18643 |  | 0.5605 | 0.454 | -9.20663 |
| 85 | 0.000 | 2.8367 | 0.046 | -8.423 | 0.000 | 2.2810 | 0.065 | -8.5416 | 3.1289 | 0.077 | -8.09239 |  | 3.1235 | 0.077 | -8.0719 |
| 86 | 4.865 | 7.2177 | 0.004 | -29.658 | 3.305 | 2.8075 | 0.047 | -18.5632 | 0.0580 | 0.810 | -25.5803 |  | 0.1086 | 0.742 | -26.909 |
| 87 | 0.827 | 0.0374 | 0.423 | -9.9658 | 1.066 | 0.0039 | 0.475 | -9.78357 | 0.7679 | 0.381 | -9.42425 |  | 0.7677 | 0.381 | -9.40243 |
| 88 | 0.886 | 0.0149 | 0.451 | -8.2891 | 1.088 | 0.0066 | 0.468 | -7.97192 | 0.8251 | 0.364 | -7.72885 |  | 0.8253 | 0.364 | -7.71272 |
| 89 | 1.000 | 0.0000 | 1.000 | 0 | 1.000 | 0.0000 | 1.000 | 0 | 0.0000 | 1.000 | 0 |  | 0.0000 | 1.000 | 0 |
| 90 | 1.000 | 0.0000 | 1.000 | 0 | 1.000 | 0.0000 | 1.000 | 0 | 0.0000 | 1.000 | 0 |  | 0.0000 | 1.000 | 0 |
| 91 | 15.185 | 26.7251 | 0.000 | -38.876 | 7.638 | 10.1539 | 0.001 | -28.2956 | 0.2322 | 0.630 | -30.7222 |  | 0.2718 | 0.602 | -34.6164 |
| 92 | 3.429 | 3.9832 | 0.023 | -14.84 | 0.994 | 0.0000 | 0.498 | -8.2743 | 2.8128 | 0.094 | -14.3118 |  | 2.6492 | 0.104 | -14.3627 |
| 93 | 2.856 | 2.9280 | 0.044 | -19.575 | 2.850 | 2.2247 | 0.068 | -16.5407 | 1.4367 | 0.231 | -16.3654 |  | 1.4479 | 0.229 | -19.2847 |
| 94 | 4.206 | 4.9835 | 0.013 | -22.29 | 4.105 | 3.5611 | 0.030 | -18.9237 | 4.3253 | 0.038 | -20.3793 |  | 4.2284 | 0.040 | -21.5925 |
| 95 | 1.000 | 0.0000 | 1.000 | 0 | 1.000 | 0.0000 | 1.000 | 0 | 0.0000 | 1.000 | 0 |  | 0.0000 | 1.000 | 0 |
| 96 | 0.632 | 0.2390 | 0.312 | -9.873 | 0.811 | 0.0457 | 0.415 | -9.5005 | 0.5565 | 0.456 | -9.35091 |  | 0.5543 | 0.457 | -9.37038 |
| 97 | 8.668 | 16.5019 | 0.000 | -28.484 | 6.499 | 9.5498 | 0.001 | -21.4388 | 3.6491 | 0.056 | -25.1353 |  | 3.9433 | 0.047 | -28.1616 |
| 98 | 0.810 | 0.0462 | 0.415 | -17.437 | 1.036 | 0.0012 | 0.486 | -17.691 | 1.8690 | 0.172 | -16.3209 |  | 1.8146 | 0.178 | -16.1719 |
| 99 | 1.000 | 0.0000 | 1.000 | 0 | 1.000 | 0.0000 | 1.000 | 0 | 0.0000 | 1.000 | 0 |  | 0.0000 | 1.000 | 0 |
| 100 | 1.000 | 0.0000 | 1.000 | 0 | 1.000 | 0.0000 | 1.000 | 0 | 0.0000 | 1.000 | 0 |  | 0.0000 | 1.000 | 0 |
| 101 | 0.000 | 1.8183 | 0.089 | -24.479 | 0.000 | 1.6659 | 0.098 | -15.1338 | 7.9574 | 0.005 | -18.0137 |  | 10.5241 | 0.001 | -18.6719 |
| 102 | 1.000 | 0.0000 | 1.000 | 0 | 1.000 | 0.0000 | 1.000 | 0 | 0.0000 | 1.000 | 0 |  | 0.0000 | 1.000 | 0 |
| 103 | 1.644 | 0.4116 | 0.261 | -17.302 | 2.140 | 0.8873 | 0.173 | -18.3152 | 0.0203 | 0.887 | -17.9059 |  | 0.0144 | 0.904 | -17.2501 |
| 104 | 1.000 | 0.0000 | 1.000 | 0 | 1.000 | 0.0000 | 1.000 | 0 | 0.0000 | 1.000 | 0 |  | 0.0000 | 1.000 | 0 |
| 105 | 7.352 | 13.6858 | 0.000 | -27.173 | 7.531 | 10.0580 | 0.001 | -27.6063 | 6.4975 | 0.011 | -29.1076 |  | 6.6251 | 0.010 | -26.5181 |
| 106 | 8.690 | 19.8979 | 0.000 | -23.776 | 5.321 | 7.7071 | 0.003 | -19.8889 | 6.0370 | 0.014 | -23.5198 |  | 7.1906 | 0.007 | -23.1778 |
| 107 | 0.000 | 2.6895 | 0.051 | -15.072 | 0.000 | 2.0687 | 0.075 | -15.6132 | ##### | 0.001 | -9.91146 |  | 10.3641 | 0.001 | -10.7946 |
| 108 | 1.566 | 0.3385 | 0.280 | -11.891 | 2.187 | 0.9541 | 0.164 | -13.2311 | 1.7284 | 0.189 | -12.6906 |  | 1.7343 | 0.188 | -11.1925 |
| 109 | 1.363 | 0.1059 | 0.372 | -11.718 | 1.684 | 0.2903 | 0.295 | -11.0064 | 0.1179 | 0.731 | -11.641 |  | 0.1291 | 0.719 | -11.6766 |
| 110 | 1.000 | 0.0000 | 1.000 | 0 | 1.000 | 0.0000 | 1.000 | 0 | 0.0000 | 1.000 | 0 |  | 0.0000 | 1.000 | 0 |
| 111 | 1.000 | 0.0000 | 1.000 | 0 | 1.000 | 0.0000 | 1.000 | 0 | 0.0000 | 1.000 | 0 |  | 0.0000 | 1.000 | 0 |
| 112 | 1.000 | 0.0000 | 1.000 | 0 | 1.000 | 0.0000 | 1.000 | 0 | 0.0000 | 1.000 | 0 |  | 0.0000 | 1.000 | 0 |
| 113 | 0.604 | 0.2913 | 0.295 | -15.612 | 0.764 | 0.0768 | 0.391 | -15.2153 | 1.5282 | 0.216 | -14.9688 |  | 1.4655 | 0.226 | -15.023 |
| 114 | 1.000 | 0.0000 | 1.000 | 0 | 1.000 | 0.0000 | 1.000 | 0 | 0.0000 | 1.000 | 0 |  | 0.0000 | 1.000 | 0 |
| 115 | 1.000 | 0.0000 | 1.000 | 0 | 1.000 | 0.0000 | 1.000 | 0 | 0.0000 | 1.000 | 0 |  | 0.0000 | 1.000 | 0 |
| 116 | 1.399 | 0.1965 | 0.329 | -11.764 | 1.850 | 0.6026 | 0.219 | -11.5749 | 0.8858 | 0.347 | -11.4523 |  | 0.8855 | 0.347 | -11.4073 |
| 117 | 1.000 | 0.0000 | 1.000 | 0 | 1.000 | 0.0000 | 1.000 | 0 | 0.0000 | 1.000 | 0 |  | 0.0000 | 1.000 | 0 |
| 118 | 1.000 | 0.0000 | 1.000 | 0 | 1.000 | 0.0000 | 1.000 | 0 | 0.0000 | 1.000 | 0 |  | 0.0000 | 1.000 | 0 |
| 119 | 12.430 | 29.0496 | 0.000 | -26.018 | 4.850 | 5.8440 | 0.008 | -16.8132 | 8.1006 | 0.004 | -25.5184 |  | 8.4208 | 0.004 | -25.4913 |
| 120 | 4.682 | 6.5749 | 0.005 | -21.983 | 1.714 | 0.4623 | 0.248 | -13.2397 | 2.1894 | 0.139 | -22.0201 |  | 2.3262 | 0.127 | -21.6662 |
| 121 | 4.927 | 7.5510 | 0.003 | -20.02 | 3.458 | 3.0274 | 0.041 | -15.9459 | 6.2657 | 0.012 | -22.2903 |  | 5.2625 | 0.022 | -19.2766 |
| 122 | 1.000 | 0.0000 | 1.000 | 0 | 1.000 | 0.0000 | 1.000 | 0 | 0.0000 | 1.000 | 0 |  | 0.0000 | 1.000 | 0 |
| 123 | 15.823 | 15.0202 | 0.000 | -39.165 | 16.798 | 11.9463 | 0.000 | -34.2685 | 2.8530 | 0.091 | -33.668 |  | 5.4073 | 0.020 | -34.8666 |
| 124 | 0.000 | 2.6564 | 0.052 | -9.8006 | 0.000 | 2.0914 | 0.074 | -9.98971 | 2.5076 | 0.113 | -9.7881 |  | 2.5025 | 0.114 | -9.63533 |
| 125 | 1.000 | 0.0000 | 1.000 | 0 | 1.000 | 0.0000 | 1.000 | 0 | 0.0000 | 1.000 | 0 |  | 0.0000 | 1.000 | 0 |
| 126 | 1.000 | 0.0000 | 1.000 | 0 | 1.000 | 0.0000 | 1.000 | 0 | 0.0000 | 1.000 | 0 |  | 0.0000 | 1.000 | 0 |
| 127 | 1.000 | 0.0000 | 1.000 | 0 | 1.000 | 0.0000 | 1.000 | 0 | 0.0000 | 1.000 | 0 |  | 0.0000 | 1.000 | 0 |
| 128 | 6.073 | 3.1796 | 0.037 | -21.864 | 3.639 | 2.5457 | 0.055 | -20.4439 | 0.3925 | 0.531 | -23.6464 |  | 0.2952 | 0.587 | -20.8606 |
| 129 | 0.747 | 0.0915 | 0.381 | -8.2732 | 0.925 | 0.0061 | 0.469 | -7.7053 | 0.7118 | 0.399 | -7.67929 |  | 0.7130 | 0.398 | -7.70332 |
| 130 | 1.000 | 0.0000 | 1.000 | 0 | 1.000 | 0.0000 | 1.000 | 0 | 0.0000 | 1.000 | 0 |  | 0.0000 | 1.000 | 0 |
| 131 | 1.230 | 0.0510 | 0.411 | -13.809 | 1.706 | 0.3217 | 0.285 | -13.1687 | 0.0217 | 0.883 | -13.7411 |  | 0.0220 | 0.882 | -13.7814 |
| 132 | 2.356 | 1.6189 | 0.102 | -13.892 | 1.006 | 0.0000 | 0.498 | -8.98424 | 1.8845 | 0.170 | -13.1093 |  | 2.0132 | 0.156 | -13.3707 |
| 133 | 0.792 | 0.0575 | 0.405 | -12.529 | 1.016 | 0.0003 | 0.494 | -11.8964 | 0.9556 | 0.328 | -11.7464 |  | 0.9538 | 0.329 | -11.7597 |
| 134 | 7.462 | 14.5047 | 0.000 | -25.581 | 3.246 | 2.7105 | 0.050 | -16.1167 | 4.6775 | 0.031 | -24.0306 |  | 4.7251 | 0.030 | -25.1386 |
| 135 | 1.670 | 0.4133 | 0.260 | -20.938 | 2.248 | 0.9589 | 0.164 | -20.3666 | 1.3958 | 0.237 | -18.0915 |  | 1.4363 | 0.231 | -19.151 |
| 136 | 1.000 | 0.0000 | 1.000 | 0 | 1.000 | 0.0000 | 1.000 | 0 | 0.0000 | 1.000 | 0 |  | 0.0000 | 1.000 | 0 |
| 137 | 1.000 | 0.0000 | 1.000 | 0 | 1.000 | 0.0000 | 1.000 | 0 | 0.0000 | 1.000 | 0 |  | 0.0000 | 1.000 | 0 |
| 138 | 1.500 | 0.2774 | 0.299 | -11.515 | 1.907 | 0.6573 | 0.209 | -10.6529 | 1.1465 | 0.284 | -11.0249 |  | 1.1474 | 0.284 | -11.0681 |
| 139 | 0.000 | 2.2588 | 0.066 | -9.7663 | 0.000 | 1.8220 | 0.089 | -9.88575 | 1.6624 | 0.197 | -9.9124 |  | 1.6597 | 0.198 | -9.7577 |
| 140 | 3.652 | 4.2748 | 0.019 | -20.1 | 2.199 | 0.9188 | 0.169 | -13.9259 | 3.1121 | 0.078 | -18.3304 |  | 3.0455 | 0.081 | -19.5633 |
| 141 | 1.000 | 0.0000 | 1.000 | 0 | 1.000 | 0.0000 | 1.000 | 0 | 0.0000 | 1.000 | 0 |  | 0.0000 | 1.000 | 0 |
| 142 | 9.194 | 18.8745 | 0.000 | -30.29 | 6.729 | 9.6874 | 0.001 | -22.9066 | 3.1522 | 0.076 | -28.8627 |  | 2.8430 | 0.092 | -30.1268 |
| 143 | 7.794 | 15.4404 | 0.000 | -22.682 | 3.728 | 3.3038 | 0.035 | -16.137 | 5.7169 | 0.017 | -22.7049 |  | 6.0182 | 0.014 | -22.1427 |
| 144 | 4.922 | 8.0805 | 0.002 | -24.413 | 4.354 | 5.0750 | 0.012 | -19.3349 | 3.2373 | 0.072 | -20.8919 |  | 3.2091 | 0.073 | -23.987 |
| 145 | 0.000 | 1.9034 | 0.084 | -21.116 | 0.000 | 1.4782 | 0.112 | -22.5093 | 9.4435 | 0.002 | -18.8515 |  | 8.2575 | 0.004 | -16.5316 |
| 146 | 1.000 | 0.0000 | 1.000 | 0 | 1.000 | 0.0000 | 1.000 | 0 | 0.0000 | 1.000 | 0 |  | 0.0000 | 1.000 | 0 |
| 147 | 1.620 | 0.3885 | 0.267 | -12.796 | 2.081 | 0.8297 | 0.181 | -12.0785 | 1.4543 | 0.228 | -12.3684 |  | 1.4523 | 0.228 | -12.2511 |
| 148 | 4.170 | 5.4737 | 0.010 | -20.13 | 4.781 | 5.6639 | 0.009 | -19.9519 | 4.6333 | 0.031 | -19.7898 |  | 4.6497 | 0.031 | -19.3608 |
| 149 | 1.737 | 0.4966 | 0.240 | -28.291 | 2.303 | 1.0441 | 0.153 | -16.5337 | 5.3531 | 0.021 | -22.5763 |  | 4.7728 | 0.029 | -22.5483 |
| 150 | 17.391 | 27.2378 | 0.000 | -31.456 | 12.072 | 18.9529 | 0.000 | -26.5409 | 5.9642 | 0.015 | -29.9193 |  | 5.4247 | 0.020 | -31.4492 |
| 151 | 1.093 | 0.0093 | 0.462 | -13.013 | 1.457 | 0.1597 | 0.345 | -12.4677 | 0.3821 | 0.537 | -12.7483 |  | 0.3790 | 0.538 | -12.7883 |
| 152 | 2.736 | 2.0972 | 0.074 | -21.54 | 3.706 | 3.1787 | 0.037 | -21.545 | 0.2252 | 0.635 | -21.9556 |  | 0.2214 | 0.638 | -21.4751 |
| 153 | 1.000 | 0.0000 | 1.000 | 0 | 1.000 | 0.0000 | 1.000 | 0 | 0.0000 | 1.000 | 0 |  | 0.0000 | 1.000 | 0 |
| 154 | 2.489 | 1.7010 | 0.096 | -15.918 | 2.873 | 2.2755 | 0.066 | -13.23 | 1.6830 | 0.195 | -12.3946 |  | 1.7596 | 0.185 | -15.4927 |
| 155 | 2.964 | 3.1716 | 0.037 | -17.345 | 3.895 | 4.6290 | 0.016 | -16.5937 | 1.6058 | 0.205 | -16 |  | 1.6012 | 0.206 | -17.0306 |
| 156 | 1.000 | 0.0000 | 1.000 | 0 | 1.000 | 0.0000 | 1.000 | 0 | 0.0000 | 1.000 | 0 |  | 0.0000 | 1.000 | 0 |
| 157 | 1.000 | 0.0000 | 1.000 | 0 | 1.000 | 0.0000 | 1.000 | 0 | 0.0000 | 1.000 | 0 |  | 0.0000 | 1.000 | 0 |
| 158 | 1.495 | 0.2706 | 0.301 | -11.769 | 1.882 | 0.6186 | 0.216 | -11.7342 | 1.0913 | 0.296 | -10.3927 |  | 1.0710 | 0.301 | -11.3548 |
| 159 | 2.748 | 2.0769 | 0.075 | -16.034 | 2.254 | 0.9610 | 0.163 | -12.677 | 2.4894 | 0.115 | -14.5018 |  | 2.4495 | 0.118 | -15.4641 |
| 160 | 5.501 | 9.5689 | 0.001 | -29.731 | 5.735 | 8.2911 | 0.002 | -27.1171 | 4.6503 | 0.031 | -28.2142 |  | 4.5319 | 0.033 | -29.173 |
| 161 | 0.776 | 0.0686 | 0.397 | -11.84 | 0.994 | 0.0000 | 0.498 | -11.2372 | 0.7371 | 0.391 | -11.2567 |  | 0.7364 | 0.391 | -11.2699 |
| 162 | 0.672 | 0.1726 | 0.339 | -13.864 | 0.846 | 0.0281 | 0.433 | -13.9204 | 1.3982 | 0.237 | -13.2859 |  | 1.4107 | 0.235 | -13.2248 |
| 163 | 1.038 | 0.0013 | 0.486 | -20.903 | 1.470 | 0.1205 | 0.364 | -15.1565 | 5.1693 | 0.023 | -16.1946 |  | 4.8743 | 0.027 | -15.6867 |
| 164 | 6.825 | 12.4643 | 0.000 | -25.152 | 11.544 | 18.3394 | 0.000 | -27.6291 | 3.9173 | 0.048 | -26.6457 |  | 3.8845 | 0.049 | -24.7812 |
| 165 | 0.700 | 0.1364 | 0.356 | -13.003 | 0.880 | 0.0162 | 0.449 | -14.3773 | 1.4831 | 0.223 | -14.0087 |  | 1.2716 | 0.259 | -12.4073 |
| 166 | 3.709 | 4.3439 | 0.019 | -19.573 | 3.534 | 3.0315 | 0.041 | -18.211 | 1.2103 | 0.271 | -22.1053 |  | 0.7541 | 0.385 | -19.5113 |
| 167 | 2.176 | 1.3781 | 0.120 | -15.299 | 2.865 | 2.3387 | 0.063 | -14.4069 | 1.9444 | 0.163 | -14.7701 |  | 1.9410 | 0.164 | -14.7637 |
| 168 | 4.164 | 5.0334 | 0.012 | -23.847 | 4.725 | 5.3403 | 0.010 | -25.209 | 0.0155 | 0.901 | -24.302 |  | 0.0080 | 0.929 | -23.4062 |
| 169 | 1.336 | 0.0000 | 0.500 | -3.5617 | 1.000 | 0.0000 | 0.500 | -3.56173 | 0.0000 | 1.000 | -3.56173 |  | 0.0000 | 1.000 | -3.56173 |
| 170 | 3.426 | 2.9756 | 0.042 | -14.09 | 5.443 | 4.9820 | 0.013 | -14.2072 | 1.0450 | 0.307 | -14.3967 |  | 1.0326 | 0.310 | -13.9189 |
| 171 | 0.000 | 2.6868 | 0.051 | -3.7747 | 0.000 | 2.1127 | 0.073 | -3.73694 | 0.0000 | 1.000 | -3.52687 |  | 0.0000 | 1.000 | -3.52687 |
| 172 | 0.000 | 2.9699 | 0.042 | -4.4973 | 0.000 | 2.3338 | 0.063 | -4.43151 | 0.0000 | 1.000 | -4.06614 |  | 0.0000 | 1.000 | -4.06614 |
| 173 | 0.000 | 2.6542 | 0.052 | -6.3979 | 0.000 | 2.0890 | 0.074 | -6.31728 | 3.1872 | 0.074 | -5.9889 |  | 3.1866 | 0.074 | -5.97931 |

^a^Yellow highlighting indicates P-value≤0.05

**TABLE I: Likelihood scores from Kaki**

| **Model** | **Log Likelihood** |
| --- | --- |
| M8a-pH | -2573.9 |
| M8a-pV | -2374.7 |
| M8-pH | -2494.5 |
| M8-pV | -2374.6 |

**TABLE J: Kaki variability & positive selection tests**

| **Variabiilty test** | **LRT** | **Chi Square P** | **Critical value  (P = 0.05)** | **Verdict** | **Test description** |
| --- | --- | --- | --- | --- | --- |
|  | 398.3 | 0.00 | 5.99 | Significant | M8a-pV versus M8a-pH (null) |
|  | 239.84 | 0.00 | 5.99 | Significant | M8-pV versus M8-pH (null) |
|  |  |  |  |  |  |
| **Positive selection** | |  |  |  |  |
| Rho variability | 0.2 | 0.65 | 5.99 | NS | M8a-pV (null) versus M8-pV |
| No rho variability | 158.66 | 0.00 | 5.99 | Significant | M8a-pH (null) versus M8-pH |

**TABLE K: Formation of hydrogen bond and salt bridge interactions across the p19 dimer interface.** The percentage occupation over the 200 ns simulation of the wild-type tomato bushy stunt (TBS) virus and all *permissible* point mutations to residues 139 and 143 that do not disrupt the overprinted MP. Side chain atoms are shown in italics. Note that the maximum percentage occupation is 200% due to the symmetry of the dimer interface.

| **Residue number** | | | | **Simulated Protein** | | | | | | | | |
| --- | --- | --- | --- | --- | --- | --- | --- | --- | --- | --- | --- | --- |
| **Donor** | | **Acceptor** | | **WT** | **R139G** | **R139W** | **E143A** | **E143S** | **E143T** | **E143K** | **E143Q** | **E143P** |
| Involve residues not mutated | | | |  |  |  |  |  |  |  |  |  |
| 122 | N | 122 | O | 199 | 199 | 199 | 199 | 199 | 199 | 199 | 199 | 199 |
| 120 | N | 124 | O | 196 | 197 | 197 | 196 | 195 | 198 | 193 | 195 | 197 |
| 124 | N | 120 | O | 199 | 199 | 199 | 196 | 198 | 199 | 198 | 198 | 199 |
| 117 | *NH1* | 132 | *NE2* | 5 | - | 5 | - | - | - | - | - | - |
| 117 | *NH2* | 132 | *NE2* | 27 | 6 | 21 | 23 | 9 | 26 | 13 | 12 | 17 |
| 117 | *NE* | 132 | *NE2* | - | - | - | - | 7 | 7 | - | 12 | - |
| 129 | *OG1* | 117 | O | - | - | - | 23 | - | - | - | - | - |
| 129 | *OG1* | 118 | O | 103 | 105 | 110 | 69 | 74 | 86 | 98 | 88 | 98 |
| 132 | *ND1* | 117 | O | 19 | 6 | - | 167 |  | 15 | 11 |  | 6 |
| 140 | *OG* | 140 | *OG* | 18 | - | 5 | 21 | - | - | - | - | 5 |
| Involve mutated residues | | | |  |  |  |  |  |  |  |  |  |
| 139 | *NH1* | 143 | *OE1* | 71 | - | - | - | - | - | - | - | - |
| 139 | *NH1* | 143 | *OE2* | 75 | - | - | - | - | - | - | - | - |
| 139 | *NE* | 143 | *OG* | - | - | - | - | 15 | - | - | - | - |
| 139 | *NH1* | 143 | O | - | - | - | - | - | - | 7 | - | - |
| 139 | *NH1* | 143 | *OE1* | - | - | - | - | - | - | - | 33 | - |
| 139 | *NH1* | 148 | *OT1* | - | - | - | - | 7 | - | - | 17 | - |
| 139 | *NH1* | 148 | *OT2* | - | - | - | - | 7 | - | - | 18 | - |
| 139 | *NH2* | 148 | *OT2* | - | - | - | - | 6 | - | - | 17 | - |
| 139 | *NH2* | 148 | *OT1* | - | - | - | - | 5 | - | - | 18 | - |
| 139 | *NH2* | 148 | *OT2* | - | - | - | - | 6 | - | - | - | - |
| 143 | *OG* | 143 | *OG* | - | - | - | - | 5 | - | - | - | - |
| 143 | *NE2* | 139 | O | - | - | - | - | - | - | - | 53 | - |
| 143 | *NE2* | 143 | *OE1* | - | - | - | - | - | - | - | 31 | - |
| 143 | *NE2* | 140 | *OG* | - | - | - | - | - | - | - | 8 | - |

**TABLE L: Numbers of atoms and dimensions of simulation boxes.**

| **System** | **Number of atoms** | | | | | | **Box dimensions (nm)** | | |
| --- | --- | --- | --- | --- | --- | --- | --- | --- | --- |
| ***Observed* (RNA*)*** | **protein** | **RNA** | **water** | **+ions** | **-ions** | **total** | **x** | **y** | **z** |
| AMC | 3906 | 1219 | 44094 | 33 | 0 | 49252 | 7.94 | 2.37 | 5.00 |
| CBL | 3988 | 1219 | 44760 | 29 | 0 | 49996 | 8.14 | 2.38 | 4.90 |
| CIR | 3900 | 1219 | 45609 | 33 | 0 | 50761 | 7.97 | 2.38 | 4.97 |
| CNV | 3964 | 1219 | 45600 | 31 | 0 | 50814 | 8.01 | 2.29 | 4.88 |
| CRV | 3922 | 1219 | 49059 | 31 | 0 | 54231 | 8.40 | 2.36 | 4.92 |
| HRV | 3906 | 1219 | 44799 | 31 | 0 | 49955 | 8.01 | 2.38 | 4.87 |
| LET | 3854 | 1219 | 46134 | 37 | 0 | 51244 | 7.89 | 2.33 | 4.82 |
| LNV | 3916 | 1219 | 49101 | 31 | 0 | 54267 | 8.39 | 2.09 | 4.98 |
| MNV | 3886 | 1219 | 43206 | 39 | 0 | 48350 | 7.83 | 2.46 | 5.04 |
| PLV | 3948 | 1219 | 45912 | 31 | 0 | 51110 | 7.90 | 2.37 | 4.93 |
| PNV | 3906 | 1219 | 44511 | 31 | 0 | 49667 | 7.93 | 2.31 | 4.96 |
| TBS | 3898 | 1219 | 48603 | 35 | 0 | 53755 | 8.46 | 2.32 | 4.95 |
| ***Observed* (no RNA)** |  |  |  |  |  |  |  |  |  |
| AMC | 3906 | 0 | 45129 | 0 | 6 | 49041 | 8.13 | 1.95 | 4.96 |
| CBL | 3988 | 0 | 43515 | 0 | 10 | 47513 | 8.18 | 1.78 | 4.97 |
| CIR | 3900 | 0 | 45855 | 0 | 6 | 49761 | 8.09 | 1.85 | 4.92 |
| CNV | 3964 | 0 | 44943 | 0 | 8 | 48915 | 8.04 | 1.89 | 4.95 |
| CRV | 3922 | 0 | 45666 | 0 | 8 | 49596 | 8.14 | 1.96 | 4.96 |
| HRV | 3906 | 0 | 44214 | 0 | 8 | 48128 | 8.07 | 1.94 | 4.96 |
| LET | 3854 | 0 | 46527 | 0 | 2 | 50383 | 8.27 | 1.93 | 4.95 |
| LNV | 3916 | 0 | 49554 | 0 | 8 | 53478 | 8.46 | 1.66 | 4.95 |
| MNV | 3886 | 0 | 43824 | 0 | 0 | 47710 | 8.10 | 1.84 | 4.96 |
| PLV | 3948 | 0 | 44301 | 0 | 8 | 48257 | 8.07 | 1.84 | 4.96 |
| PNV | 3906 | 0 | 40500 | 0 | 8 | 44414 | 7.97 | 1.78 | 4.95 |
| TBS | 3898 | 0 | 47160 | 0 | 4 | 51062 | 8.41 | 1.97 | 4.96 |
| ***Permissible* (RNA)** |  |  |  |  |  |  |  |  |  |
| WT (dimer) | 3820 | 1219 | 45903 | 37 | 0 | 50979 | 7.94 | 2.16 | 4.94 |
| WT (monomer) | 1910 | 1219 | 45360 | 38 | 0 | 48527 | 7.77 | 2.54 | 4.91 |
| R139G | 3786 | 1219 | 45642 | 39 | 0 | 50686 | 7.92 | 2.23 | 4.99 |
| R139W | 3820 | 1219 | 45657 | 39 | 0 | 50735 | 7.91 | 2.19 | 4.96 |
| E143A | 3810 | 1219 | 45771 | 35 | 0 | 50835 | 7.91 | 2.15 | 4.94 |
| E143S | 3812 | 1219 | 45846 | 35 | 0 | 50912 | 7.92 | 2.15 | 4.89 |
| E143T | 3818 | 1219 | 45762 | 35 | 0 | 50834 | 7.93 | 2.10 | 4.92 |
| E143K | 3834 | 1219 | 45453 | 33 | 0 | 50539 | 7.96 | 2.22 | 4.94 |
| E143Q | 3824 | 1219 | 46128 | 35 | 0 | 51206 | 7.93 | 2.12 | 4.94 |
| E143P | 3818 | 1219 | 45402 | 35 | 0 | 50474 | 7.90 | 2.19 | 4.95 |
| ***Permissible* (no RNA)** |  |  |  |  |  |  |  |  |  |
| WT (dimer) | 3820 | 0 | 45144 | 0 | 2 | 48966 | 7.98 | 1.88 | 4.94 |
| R139G | 3786 | 0 | 45663 | 0 | 0 | 49449 | 7.99 | 1.87 | 4.96 |
| R139W | 3820 | 0 | 45423 | 0 | 0 | 49243 | 7.94 | 1.82 | 4.96 |
| E143A | 3810 | 0 | 46101 | 0 | 4 | 49915 | 7.98 | 1.76 | 4.95 |
| E143S | 3812 | 0 | 45840 | 0 | 4 | 49656 | 8.03 | 1.82 | 4.95 |
| E143T | 3818 | 0 | 45744 | 0 | 4 | 49566 | 7.99 | 1.80 | 4.95 |
| E143K | 3834 | 0 | 45633 | 0 | 6 | 49473 | 7.97 | 1.85 | 4.95 |
| E143Q | 3824 | 0 | 46719 | 0 | 4 | 50547 | 7.99 | 1.75 | 4.96 |
| E143P | 3818 | 0 | 45699 | 0 | 4 | 49521 | 7.99 | 1.83 | 4.95 |

###
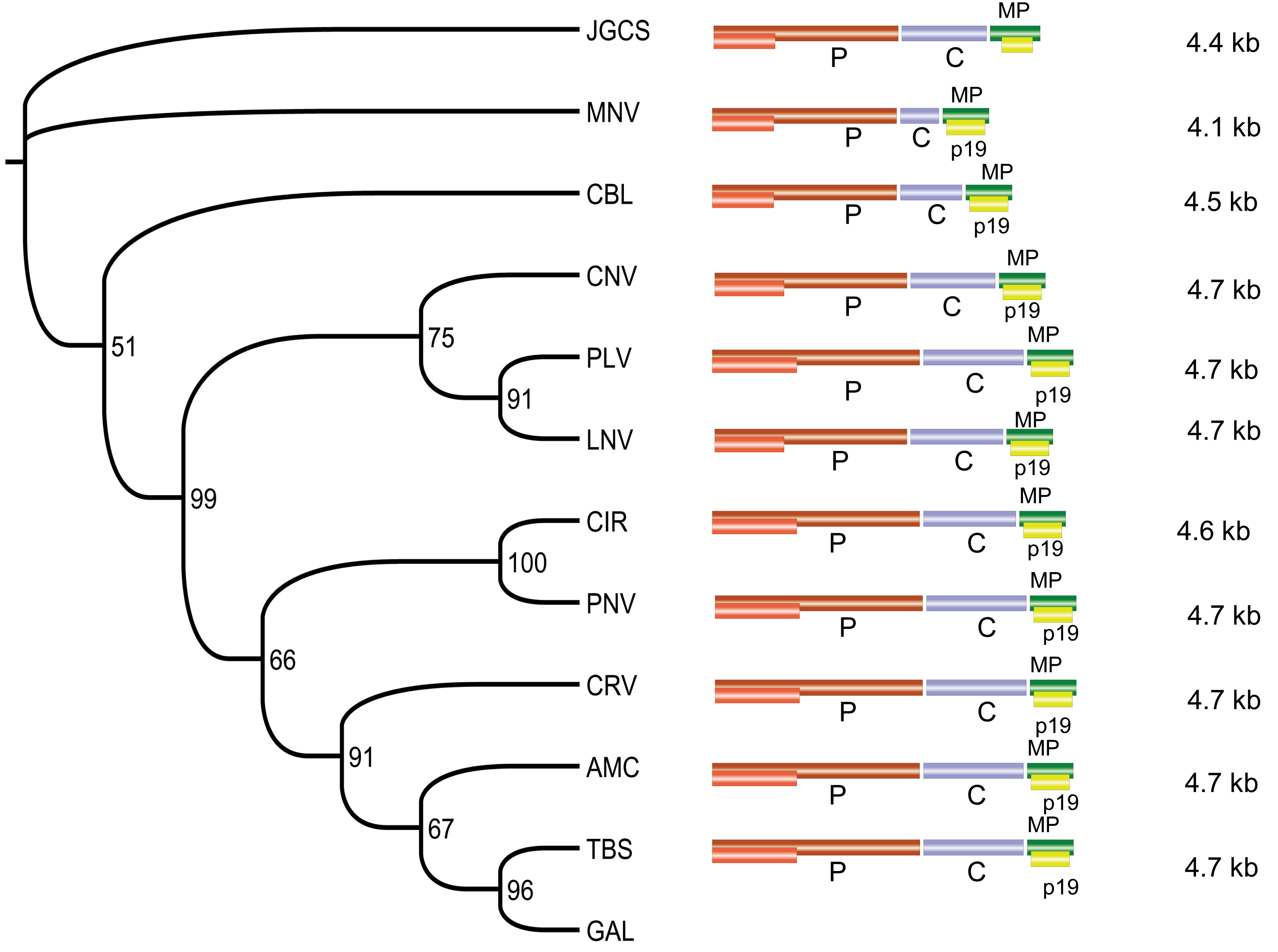


**Figure A: ML guide tree used for the PAML analysis, based on RDRP sequences from Tombusviruses.** The model of sequence evolution (GTR-G) was determined with Modeltest (Posada et al, 2006). Tree reconstruction was performed with PhyML. Maximum Parsimony and Neighbor-Joining trees (MEGA3) displayed equivalent topology to the ML tree (data not shown). Cartoons are schematics showing genome structure among tombusviruses (scaled). Note that MP and p19 are overprinted on the same DNA strand. RDRP: RNA-dependent RNA-polymerase; Coat: coat protein; MP: movement protein; p19: viral repressor of RNAi. Abbreviations for Tombusviruses in alphabetical order: AMC (Artichoke mottle crinkle virus, NC001339), CIR (Carnation italian ringspot virus, NC003500), CBL (Cucumber bulgarian latent virus, NC004725), CNV (Cucumber necrosis virus, NC001469), CRV (Cymbidium ringspot virus, NC003532), GAL (Grapevine algerian latent virus, AY830918),HRV (Havel river tombusvirus, AY370535) , LET (Lettuce necrotic stunt virus, AJ288915), LNV (Lisianthus necrosis virus, NC007983), MNV (Maize necrotic streak virus, NC007729), PLV (Pear latent virus, NC004723), PNV (Pelagornium necrotic streak virus, NC005285), TBS (Tomato bushy stunt virus, NC001554), Outgroup: JGCS (Johnsongrass chlorotic stripe mosaic virus, Aureusviruses, NC005287).


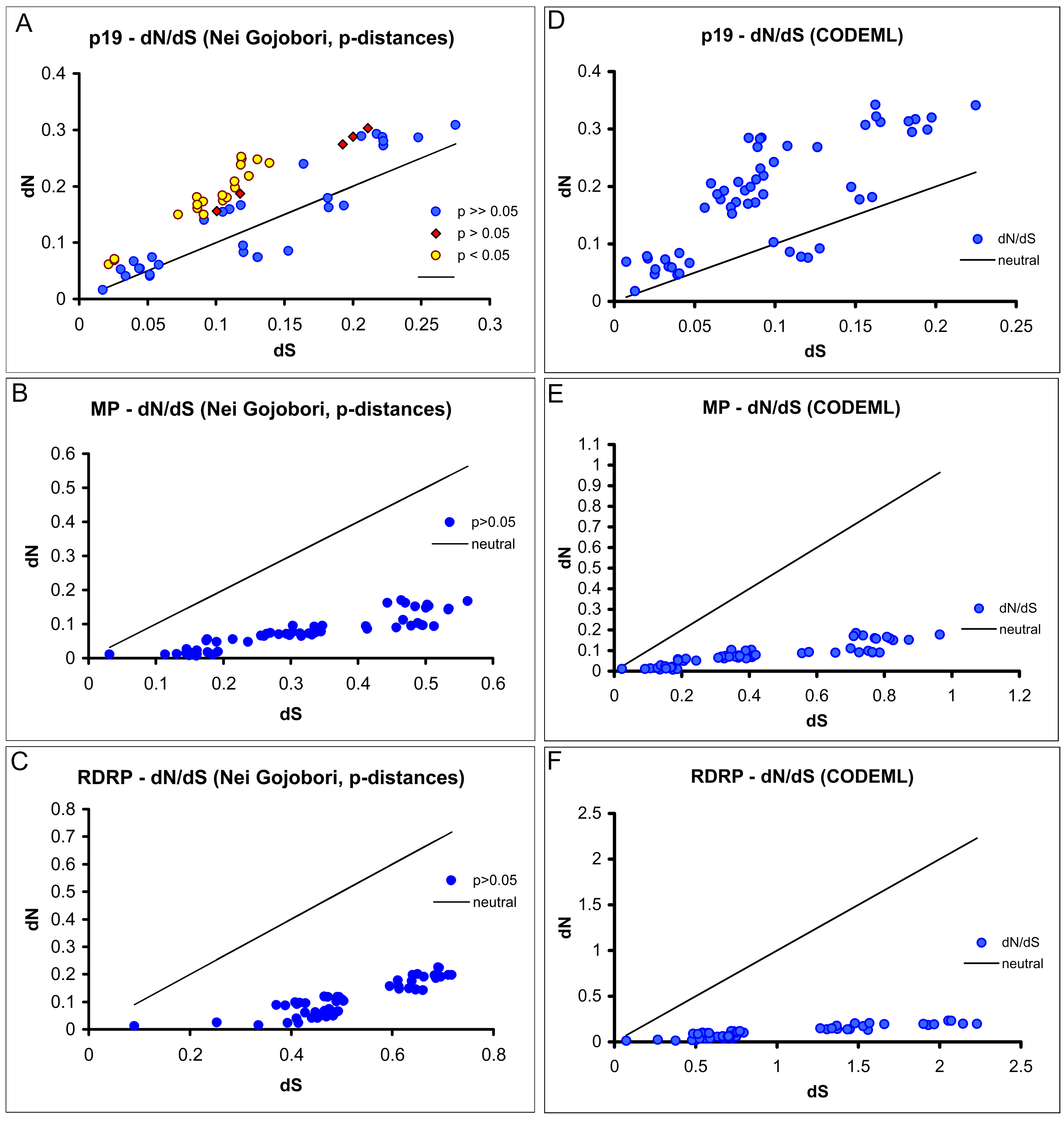


**Figure B: dN/dS scatterplots. (**A-C) dN/dS scatterplot for p19, MP and RDRP genes from 11 different tombusvirus species, based on pairwise distances (MEGA, p-distances). While both MP and RDRP exhibit clear signs of purifying selection (dN/dS < 1), the majority of p19 pairs strongly suggest positive selection (dN/dS > 1). Pairs with a dN/dS ratio significantly greater than 1 are marked in yellow, pairs only slightly above the threshold of p=0.05 are marked red. The remaining pairs do not support positive selection. (D-F) A similar comparison performed on the basis of ML estimates using codeml (runmode -2) delivered almost identical results. Here, the support for positive selection acting on p19 genes was even stronger.


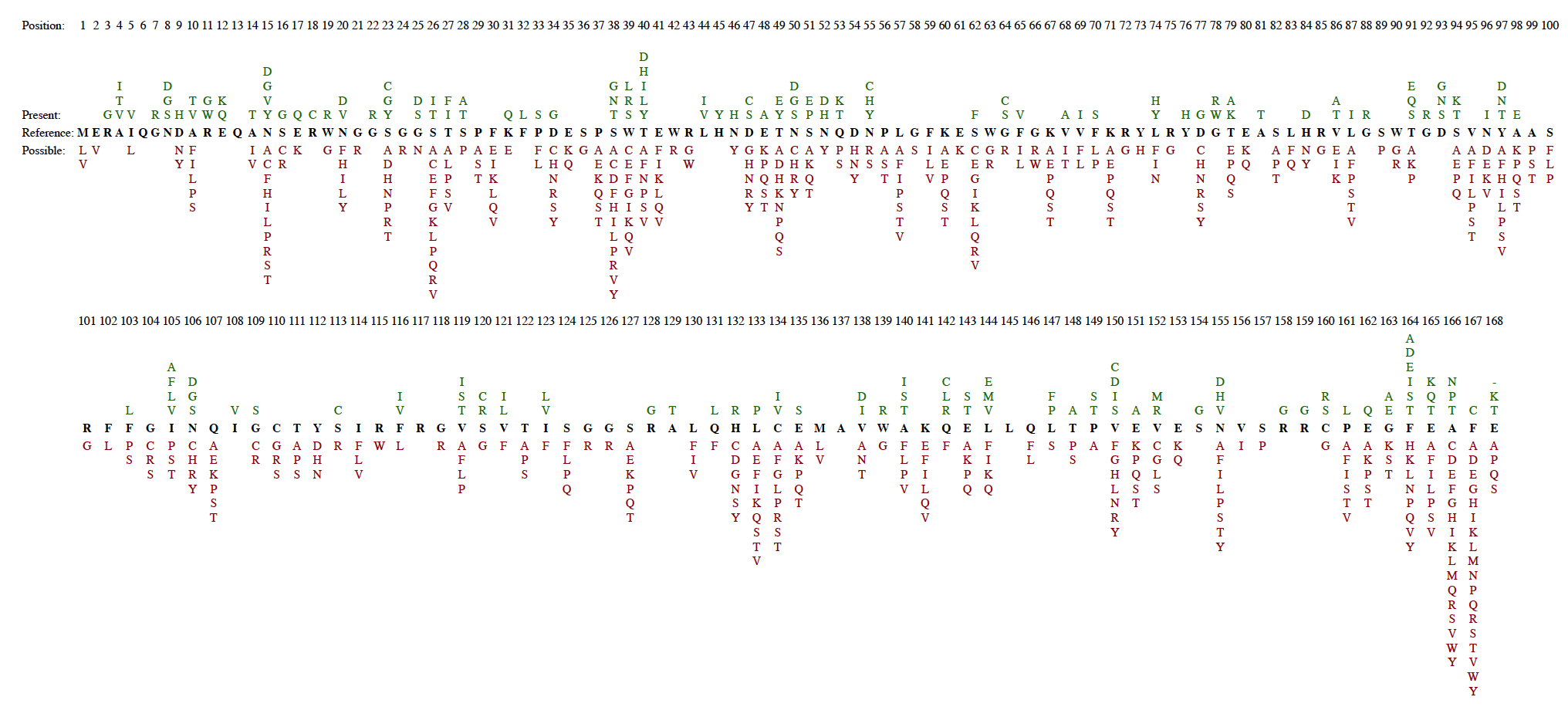


**Figure C: Observed and permissible sequence variation.** Sequence of the tomato bushy stunt (TBS) tombusvirus p19 protein (reference, black); (green, above) all sequences *observed* in known tombusvirus sequences and (red, below) all *permissible* mutations that do not disrupt the overprinted movement protein.

**Structural stability of all p19 variants studied with and without a 19 bp siRNA bound by molecular dynamics simulations.**

Figures D-O refer to all *observed* sequence variants, for which the initial structure was generated by homology modeling using the TBS p19 crystal structure as a template.

Figures P-Y refer to the wild-type tomato bushy stunt (TBS) virus p19 sequence plus the set of all *permissible* mutations to residues 139 and 143 that do not disrupt the overprinted MP generated by in silico mutation of the TBS p19 crystal structure.

For figures D-Y, the following results are presented:

(top row panels) Root-mean-square fluctuations (rmsf) of the Cα atoms of (black) subunit A and (red) subunit B.

(second row panels) Root-mean-square deviation (rmsd) of the Cα atoms from their initial positions: (black) superposition of the Cα atoms of subunit A, rmsd of the Cα atoms of subunit B; (red) superposition of the Cα atoms of subunit B, rmsd of the Cα atoms of subunit A; (green) superposition and rmsd of the Cα atoms of both subunits.

(third row panels) Secondary structure of the protein subunits during the course of the simulation: (black) 3_10_-helix; (red) coil; (green) extended; (magenta) turn; (blue) α-helix; (cyan) β-sheet; (yellow) π-helix. A white line separates subunits A (below) and B (above).

(bottom row panels) Solvent-accessible surface area (SASA) of each amino acid residue and nucleotide through the course of the simulation: (maroon) 0-1 Å^2^; (red) 1-2 Å^2^; (orange) 2-4 Å^2^; (yellow) 4-6 Å^2^; (green) 6-8 Å^2^; (cyan) 8-10 Å^2^; (blue) 10-12 Å^2^; (indigo) >12 Å^2^. White lines separate the two protein subunits and the RNA.

**
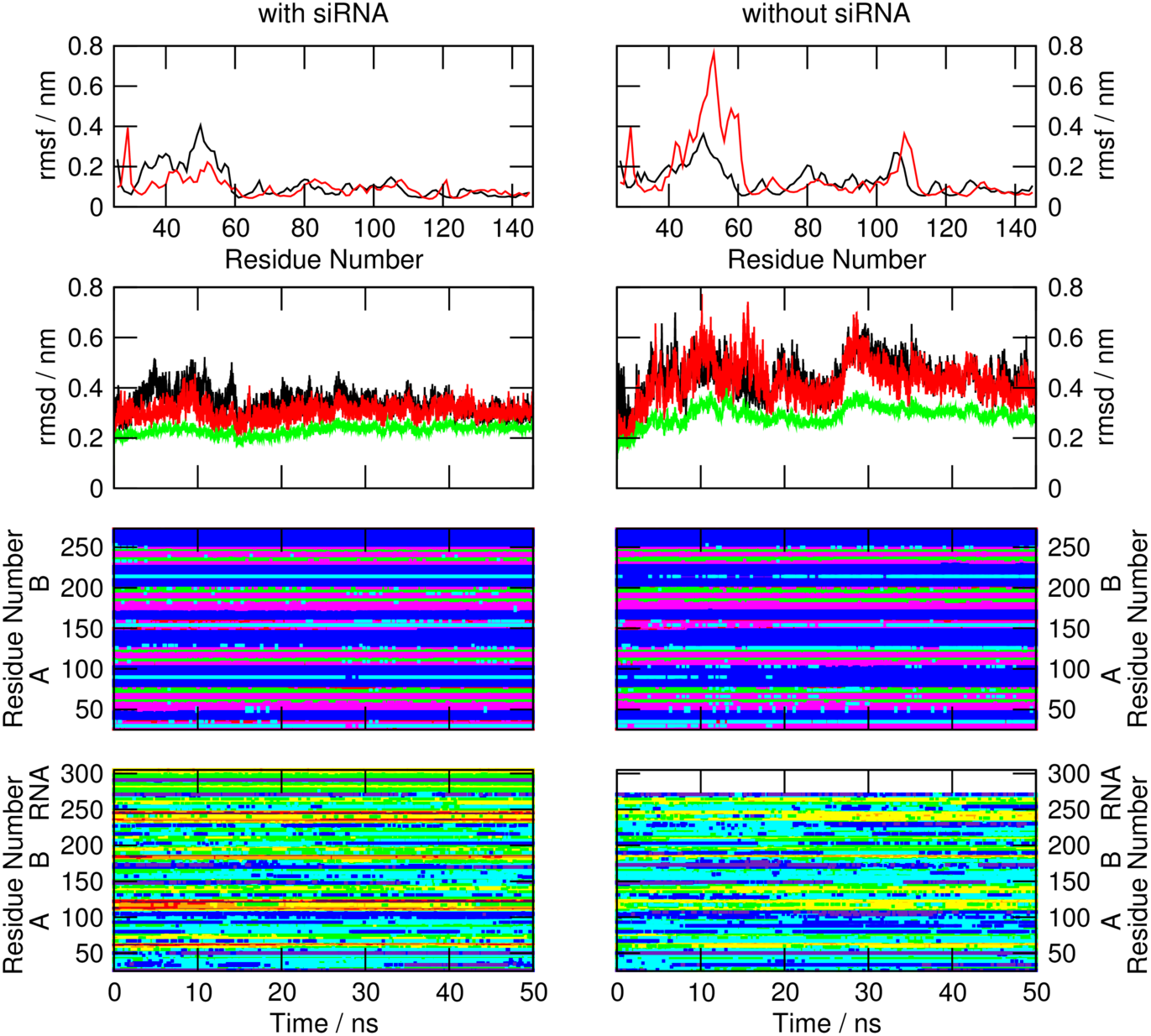
**

**Figure D:** Structural stability of the *observed* AMC (Artichoke mottle crinkle virus, NC001339) p19 sequence with and without a 19 bp siRNA bound.

**
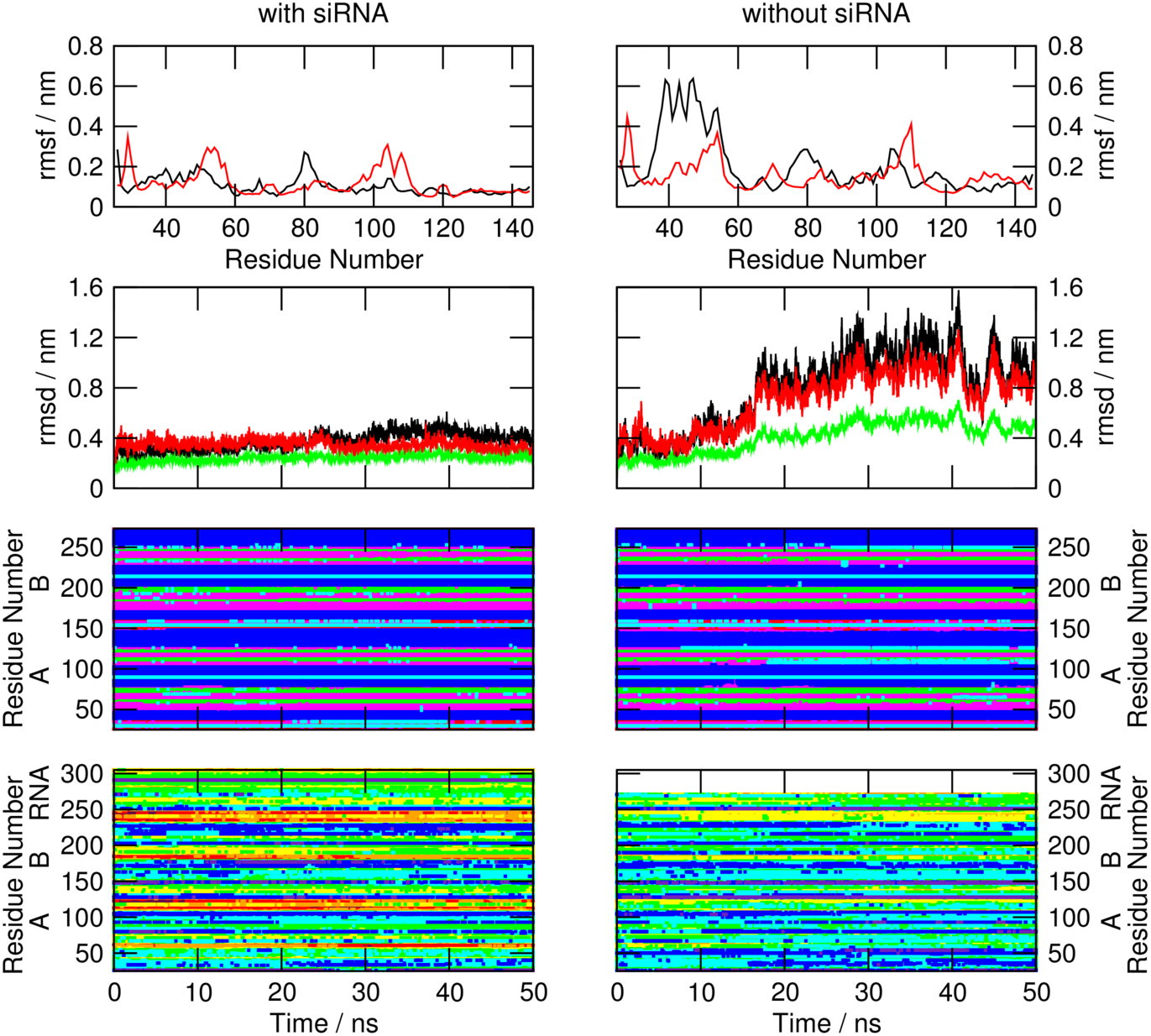
**

**Figure E:** Structural stability of the *observed* CBL (Cucumber bulgarian latent virus, NC004725) p19 sequence with and without a 19 bp siRNA bound.


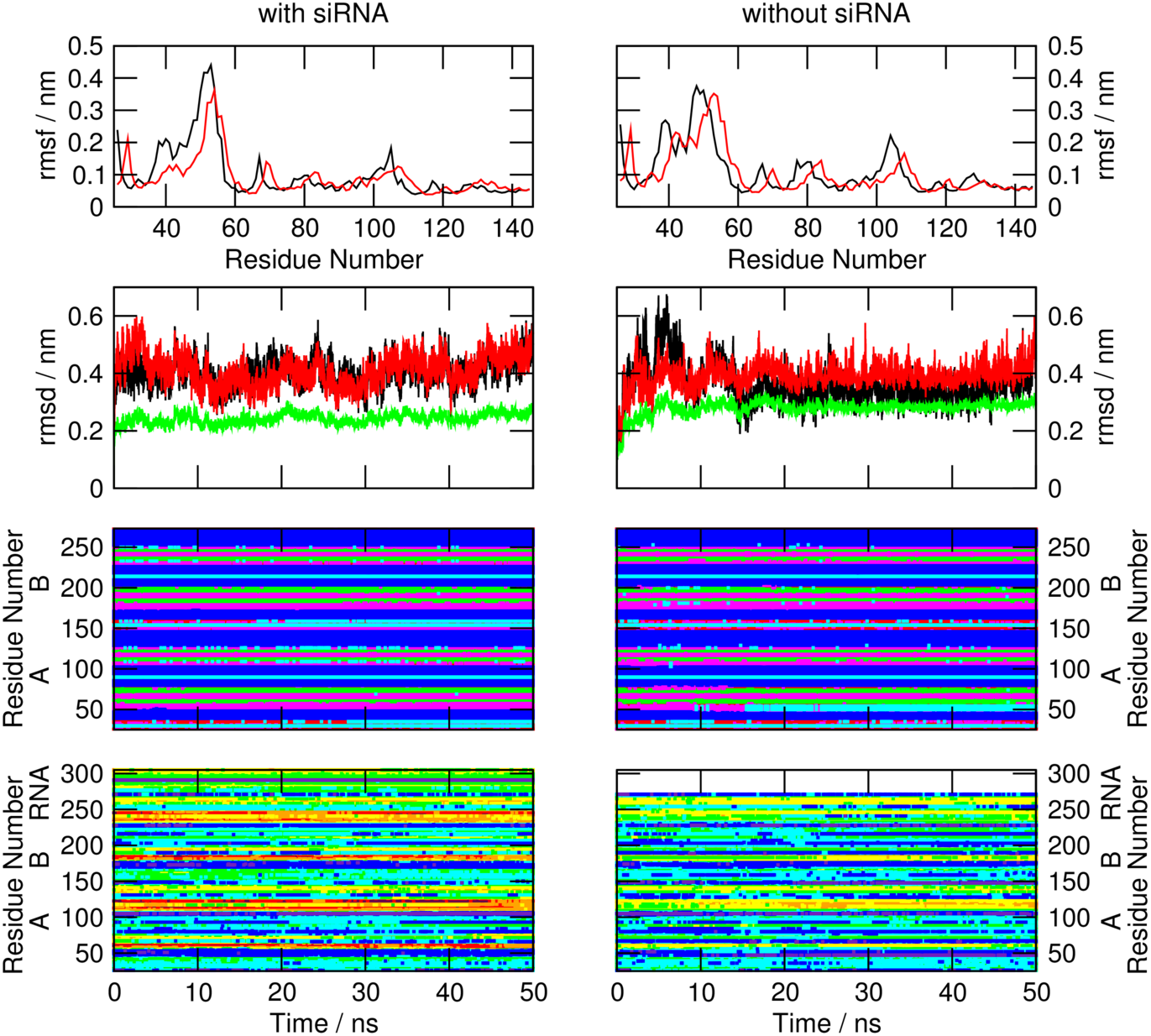


**Figure F:** Structural stability of the *observed* CIR (Carnation italian ringspot virus, NC003500) p19 sequence with and without a 19 bp siRNA bound.

**
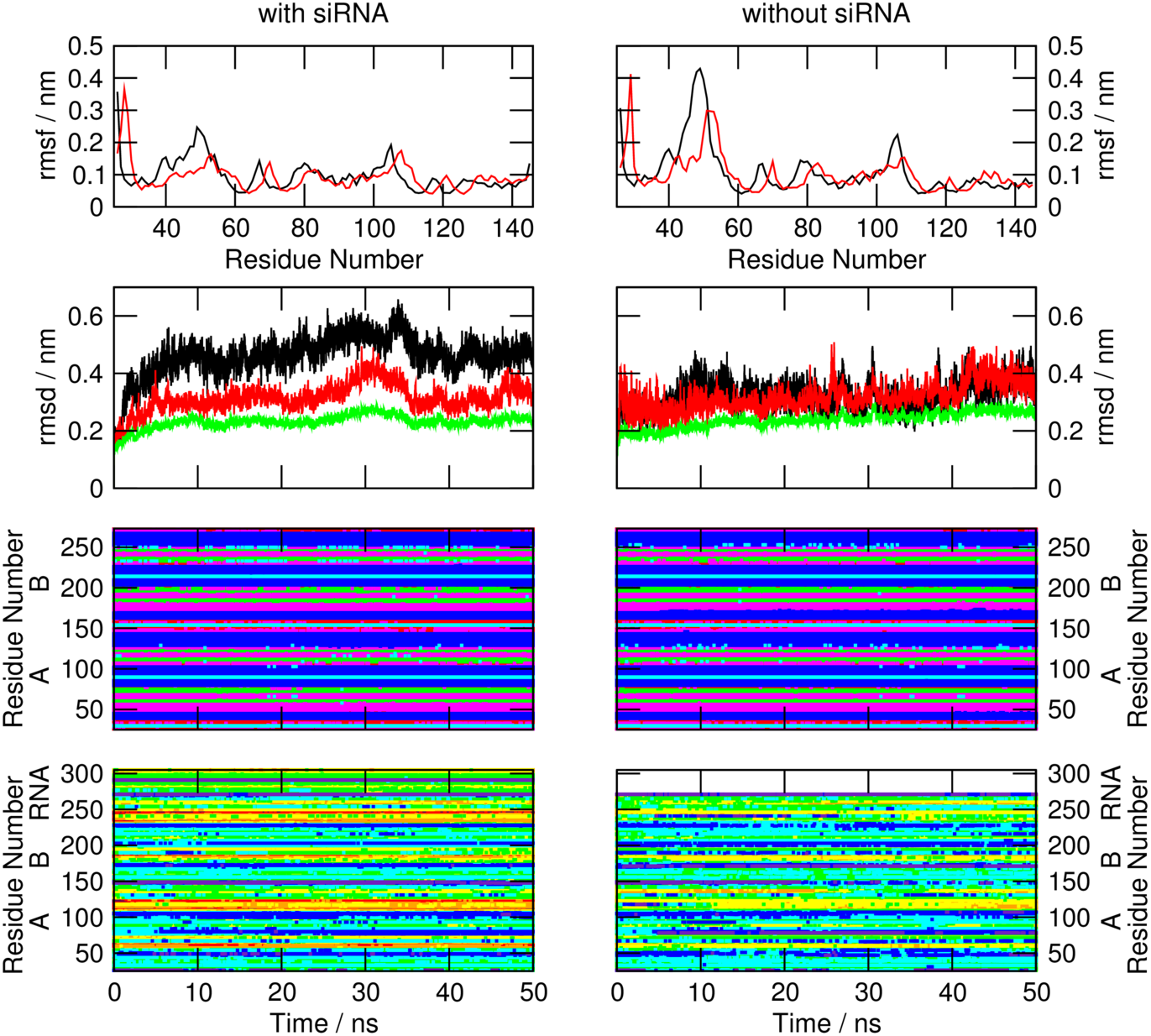
**

**Figure G:** Structural stability of the *observed* CNV (Cucumber necrosis virus, NC001469) p19 sequence with and without a 19 bp siRNA bound.

**
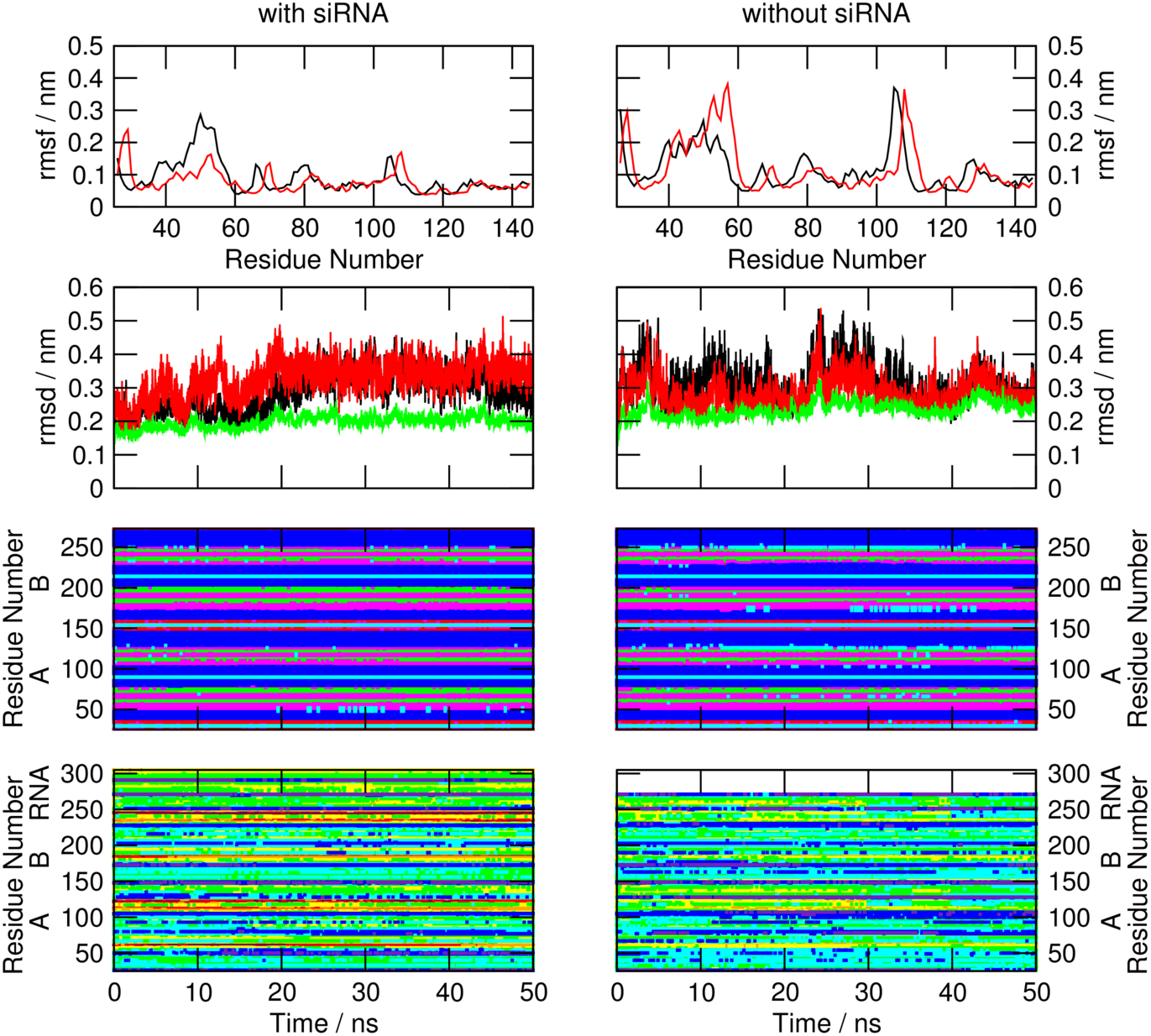
**

**Figure H:** Structural stability of the *observed* CRV (Cymbidium ringspot virus, NC003532) p19 sequence with and without a 19 bp siRNA bound.

**
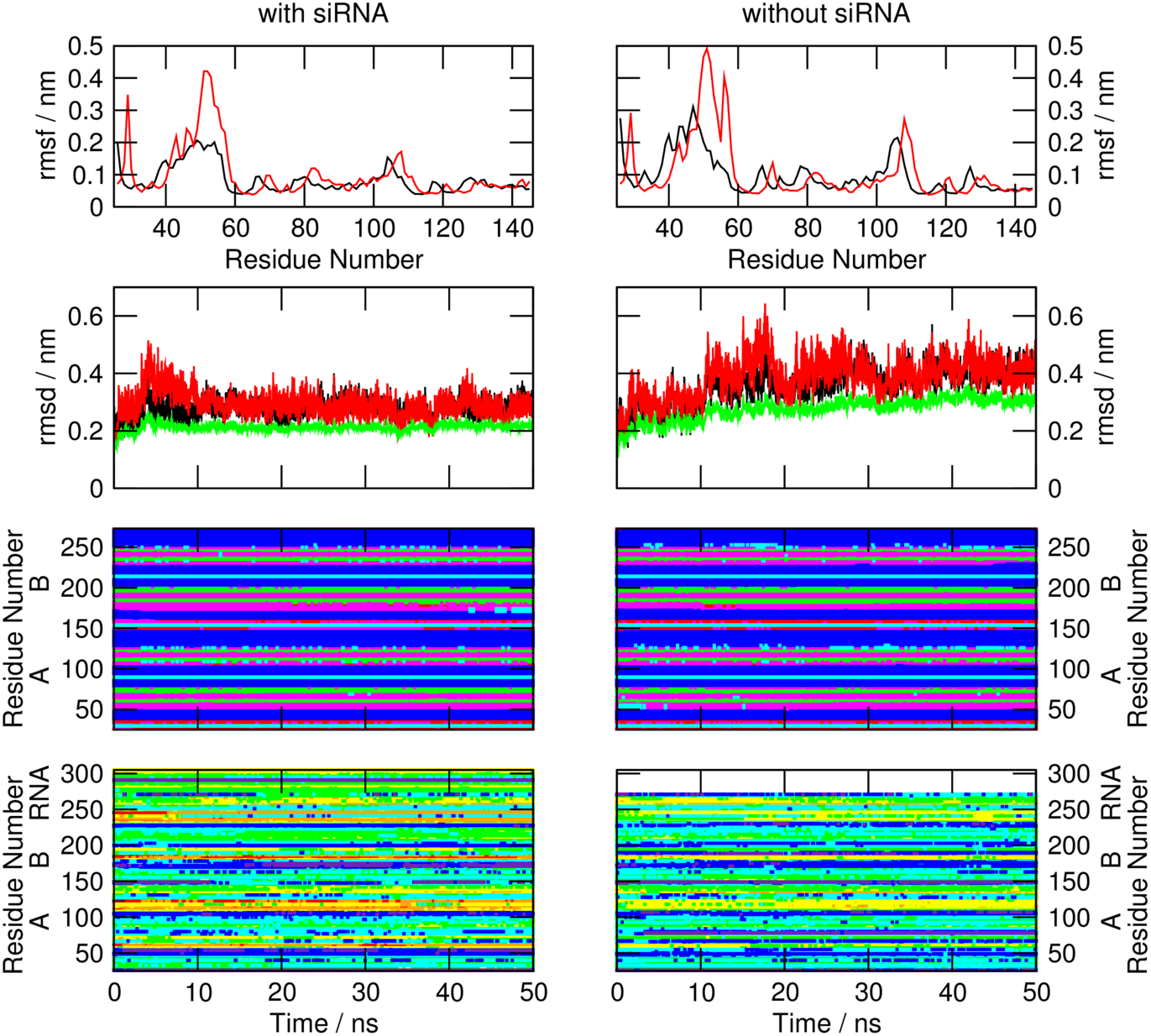
**

**Figure I:** Structural stability of the *observed* HRV (Havel river tombusvirus, AY370535) p19 sequence with and without a 19 bp siRNA bound.

**
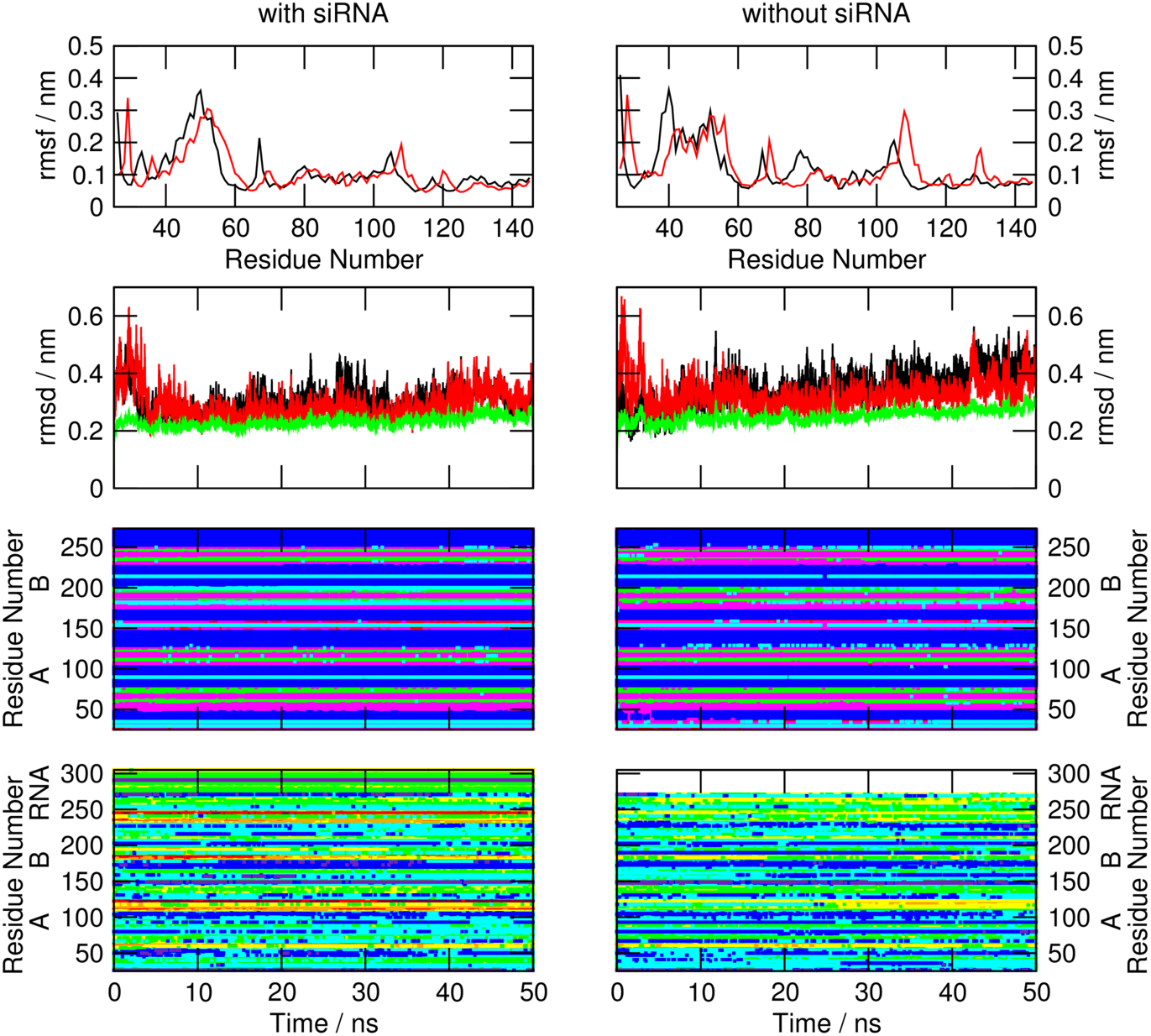
**

**Figure J:** Structural stability of the *observed* LET (Lettuce necrotic stunt virus, AJ288915) p19 sequence with and without a 19 bp siRNA bound.

**
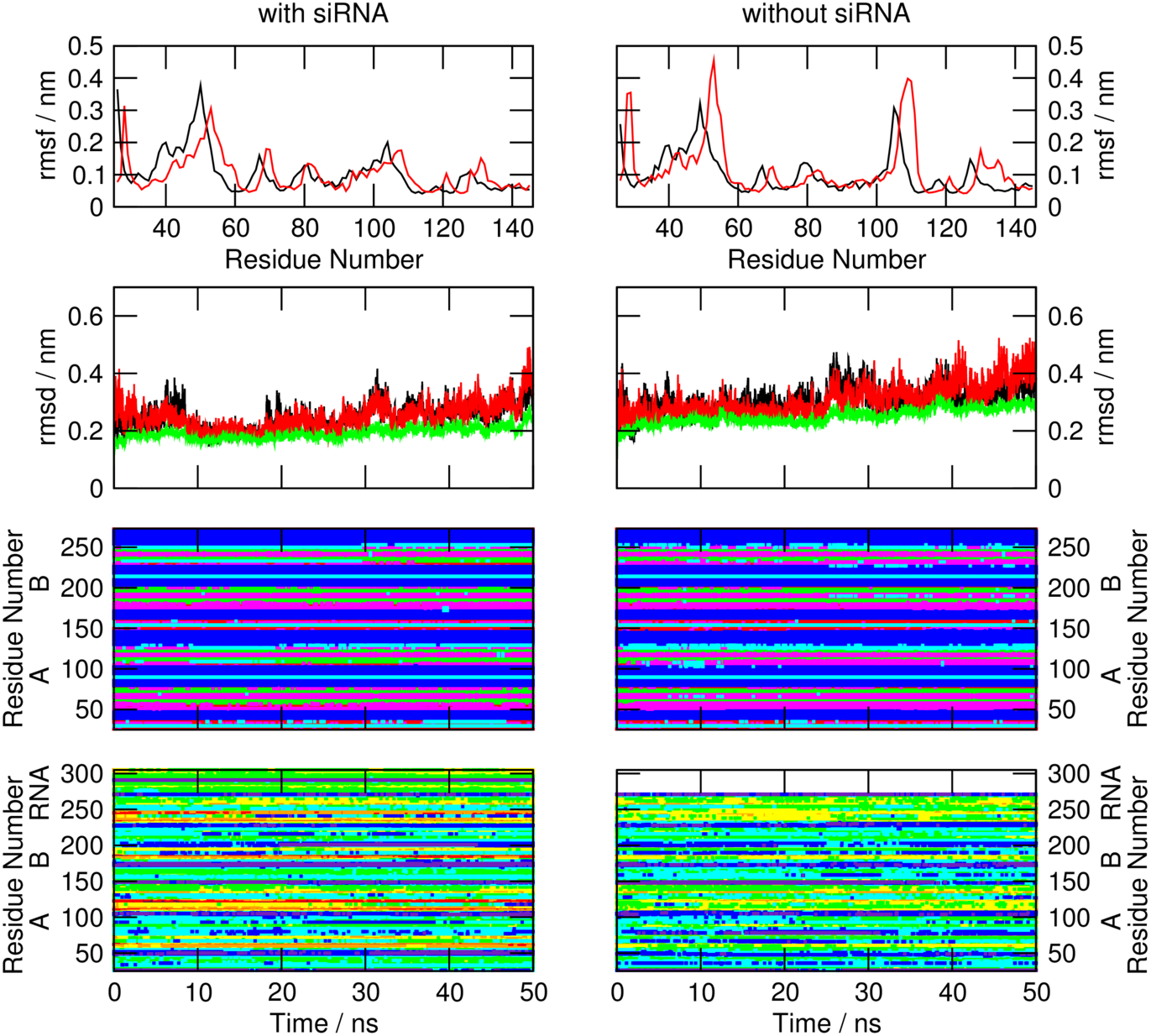
**

**Figure K:** Structural stability of the *observed* LNV (Lisianthus necrosis virus, NC007983) p19 sequence with and without a 19 bp siRNA bound.

**
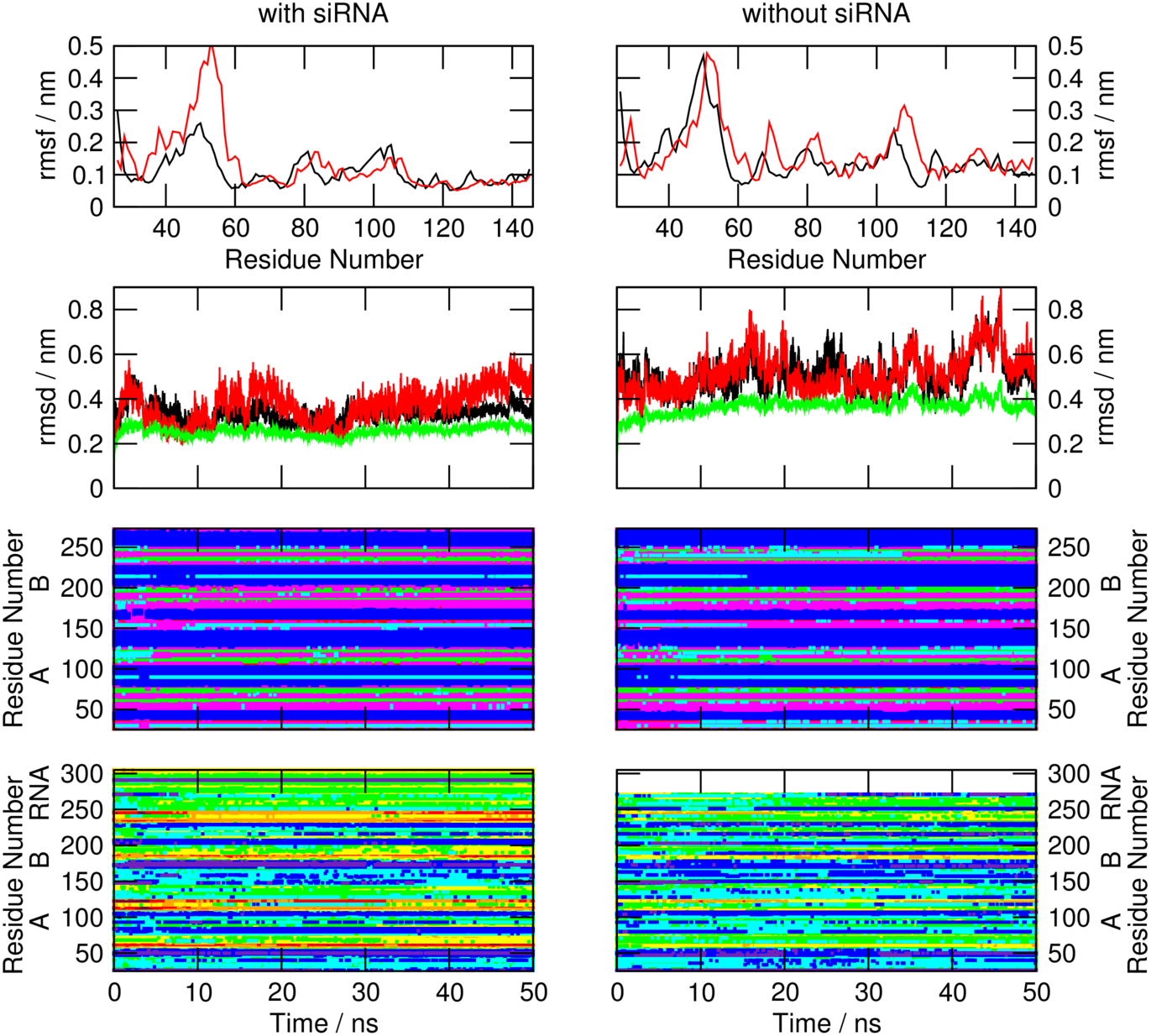
**

**Figure L:** Structural stability of the *observed* MNV (Maize necrotic streak virus, NC007729) p19 sequence with and without a 19 bp siRNA bound.

**
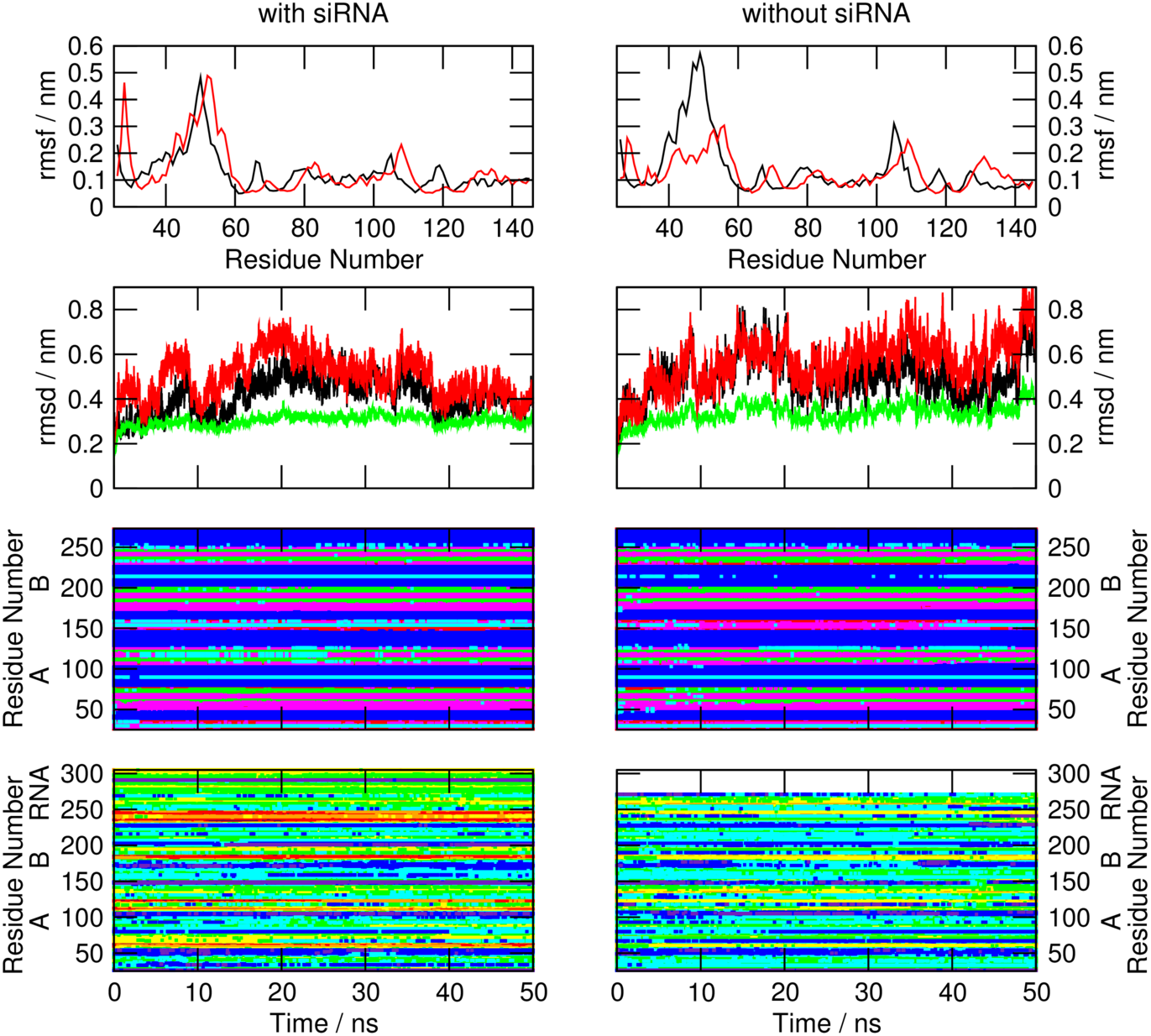
**

**Figure M:** Structural stability of the *observed* PLV (Pear latent virus, NC004723) p19 sequence with and without a 19 bp siRNA bound.

**
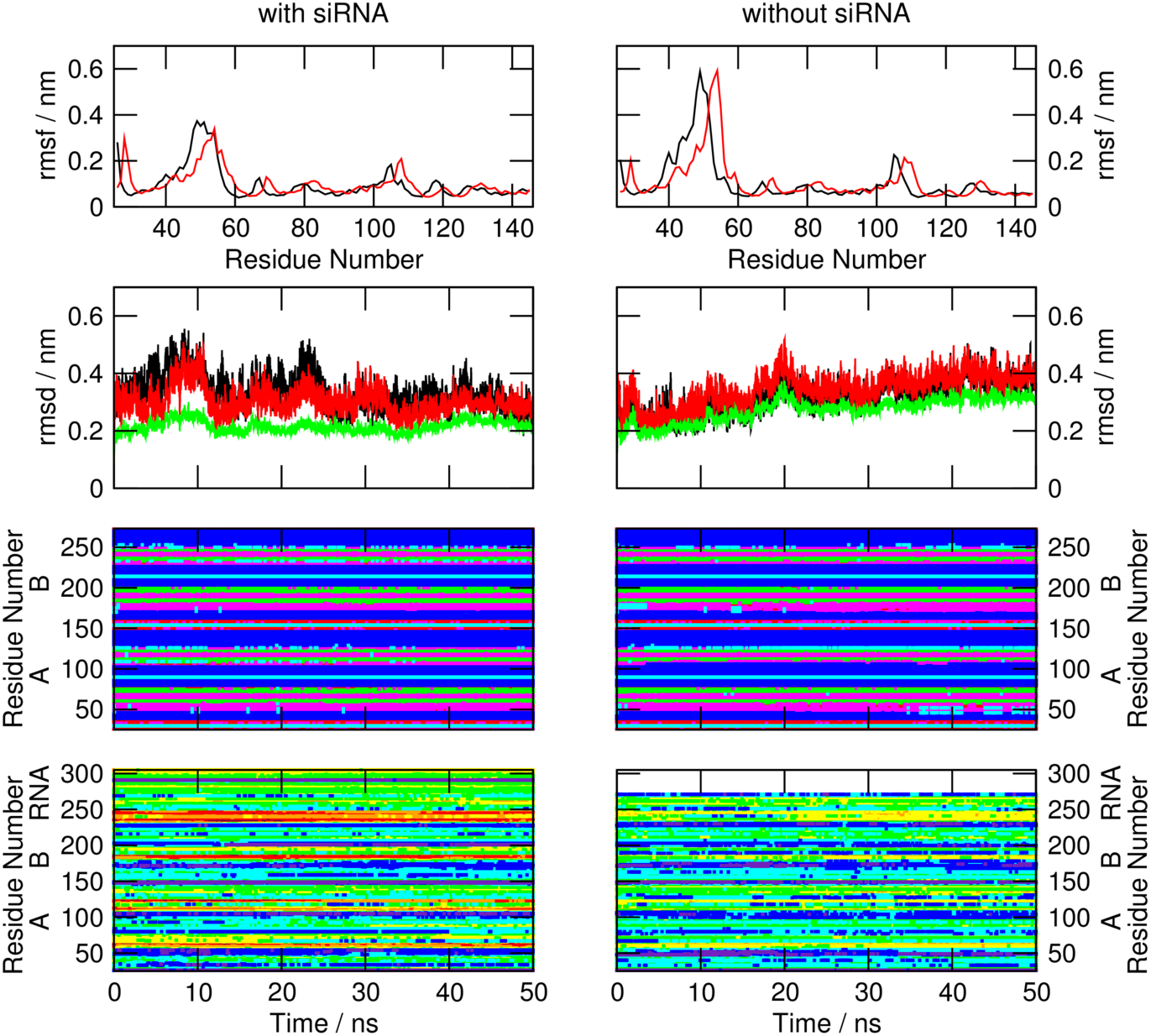
**

**Figure N:** Structural stability of the *observed* PNV (Pelagornium necrotic streak virus, NC005285) p19 sequence with and without a 19 bp siRNA bound.

**
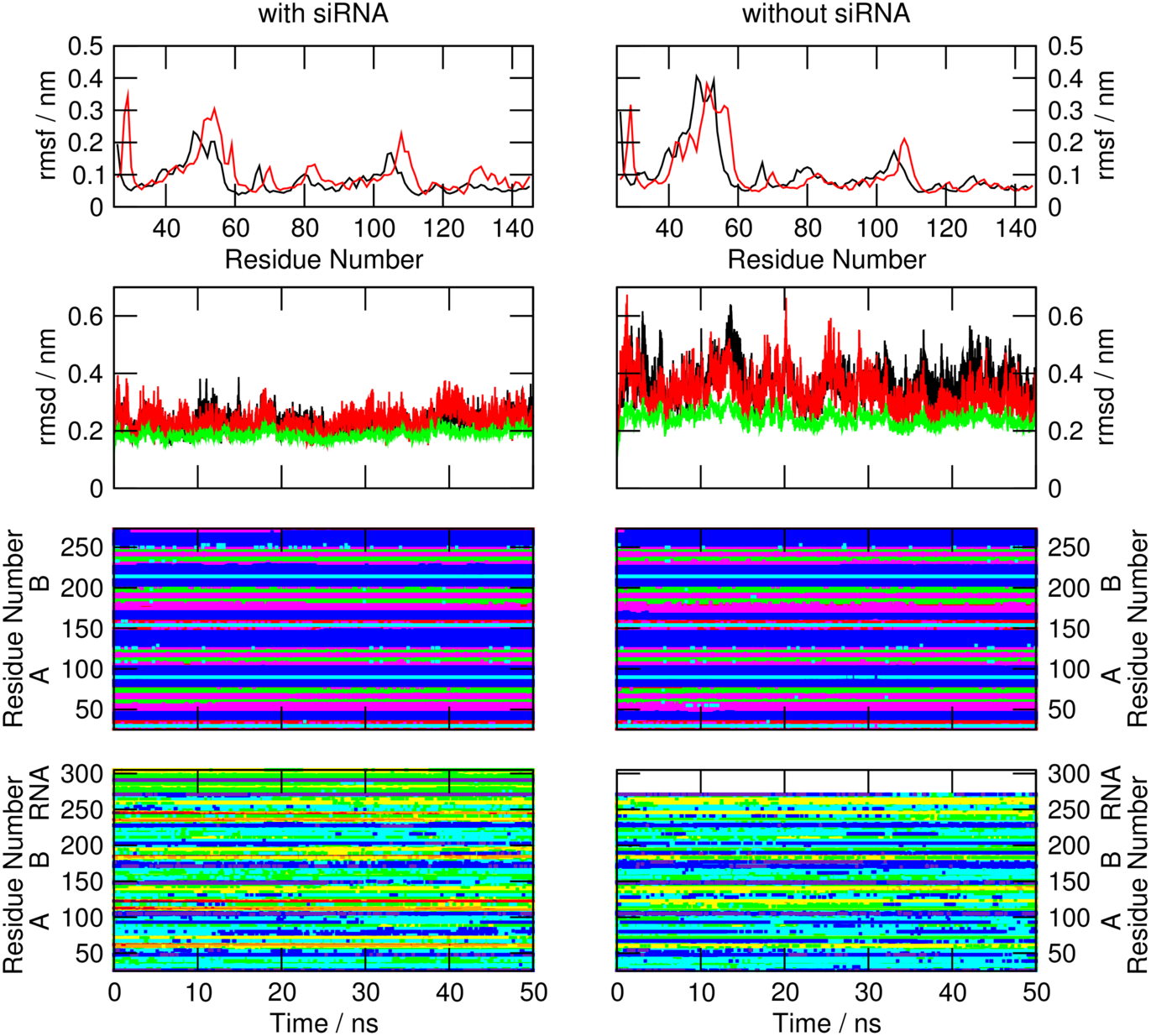
**

**Figure O:** Structural stability of the *observed* TBS (Tomato bushy stunt virus, NC001554) p19 sequence with and without a 19 bp siRNA bound.

**
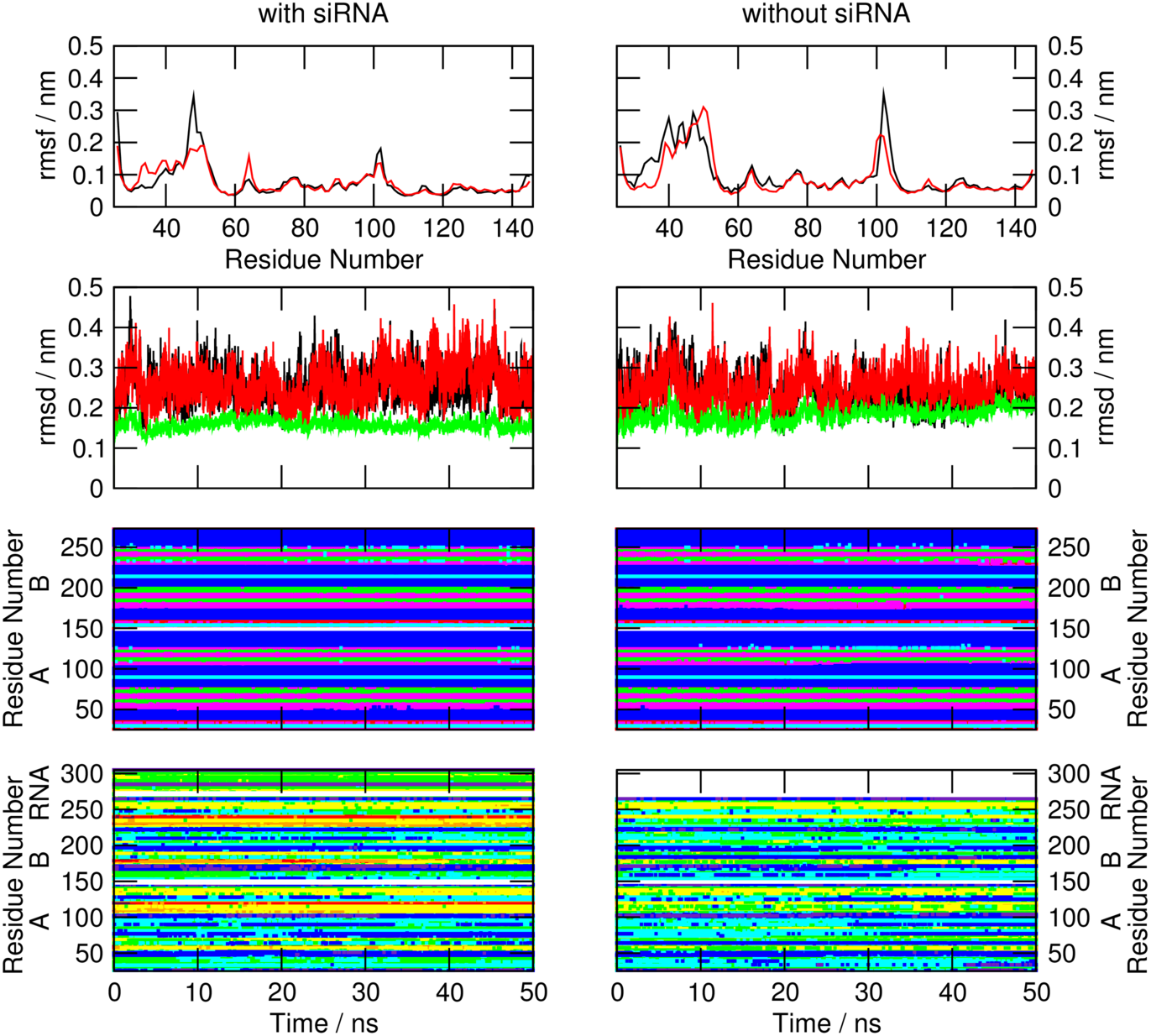
**

**Figure P:** Structural stability of the wild-type tomato bushy stunt (TBS) virus p19 sequence (PDB ID 1R9F) with and without a 19 bp siRNA bound**.**

**
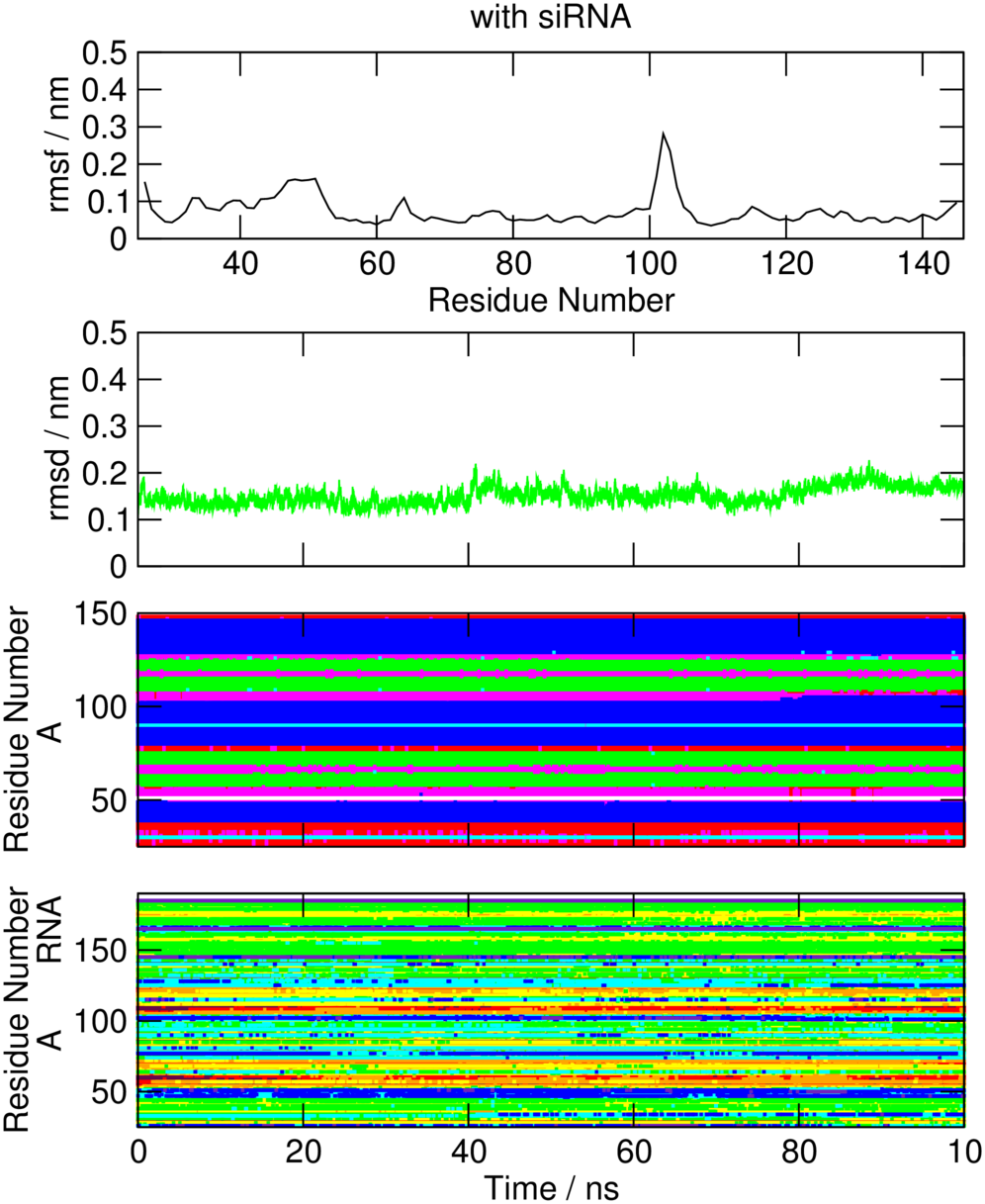
**

**Figure Q:** Structural stability of monomeric wild-type tomato bushy stunt (TBS) virus p19 sequence (PDB ID 1R9F) with and without a 19 bp siRNA bound**.**

**
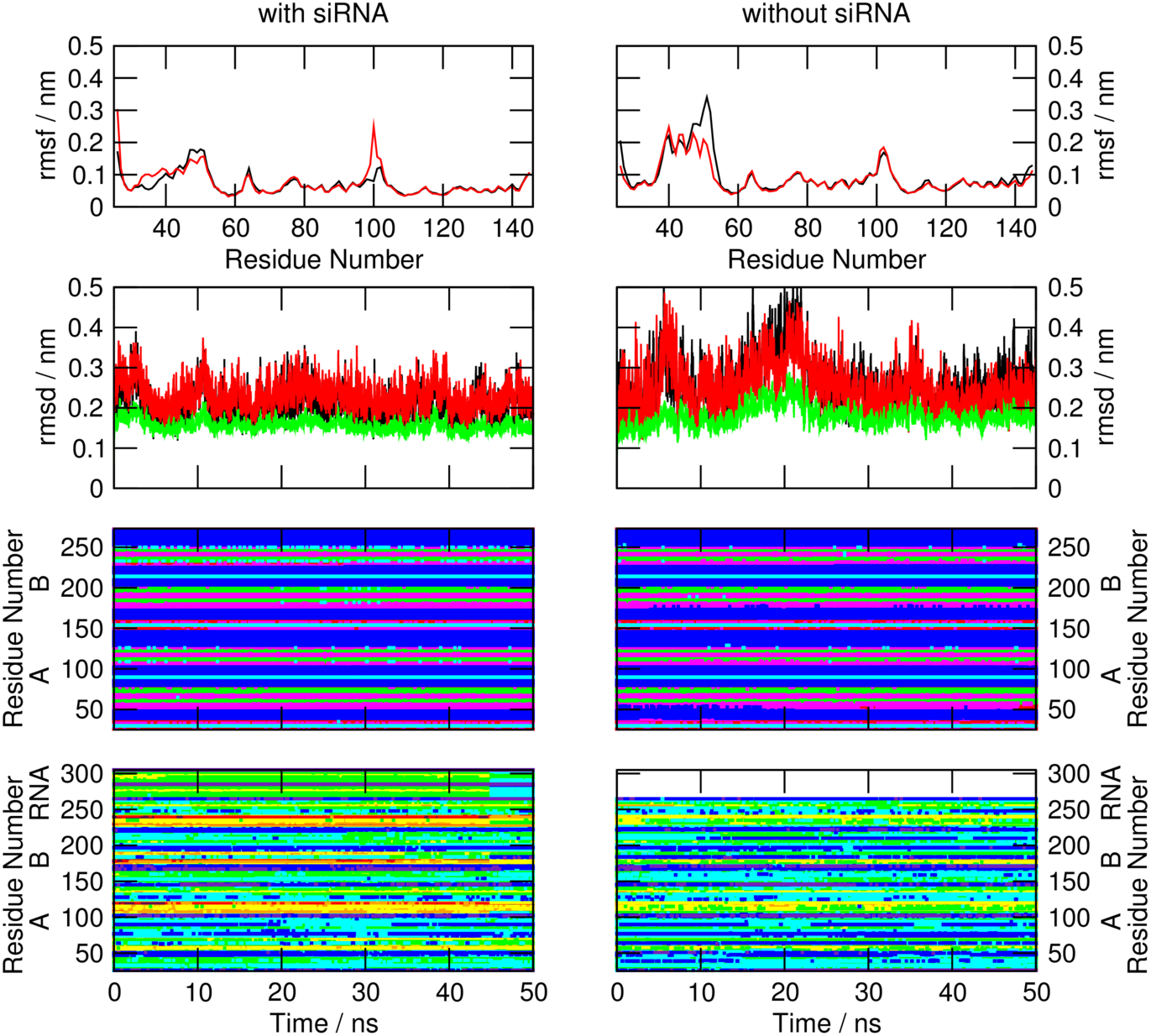
**

**Figure R:** Structural stability of the *permissible* p19 sequence variant R139G with and without a 19 bp siRNA bound**.**

**
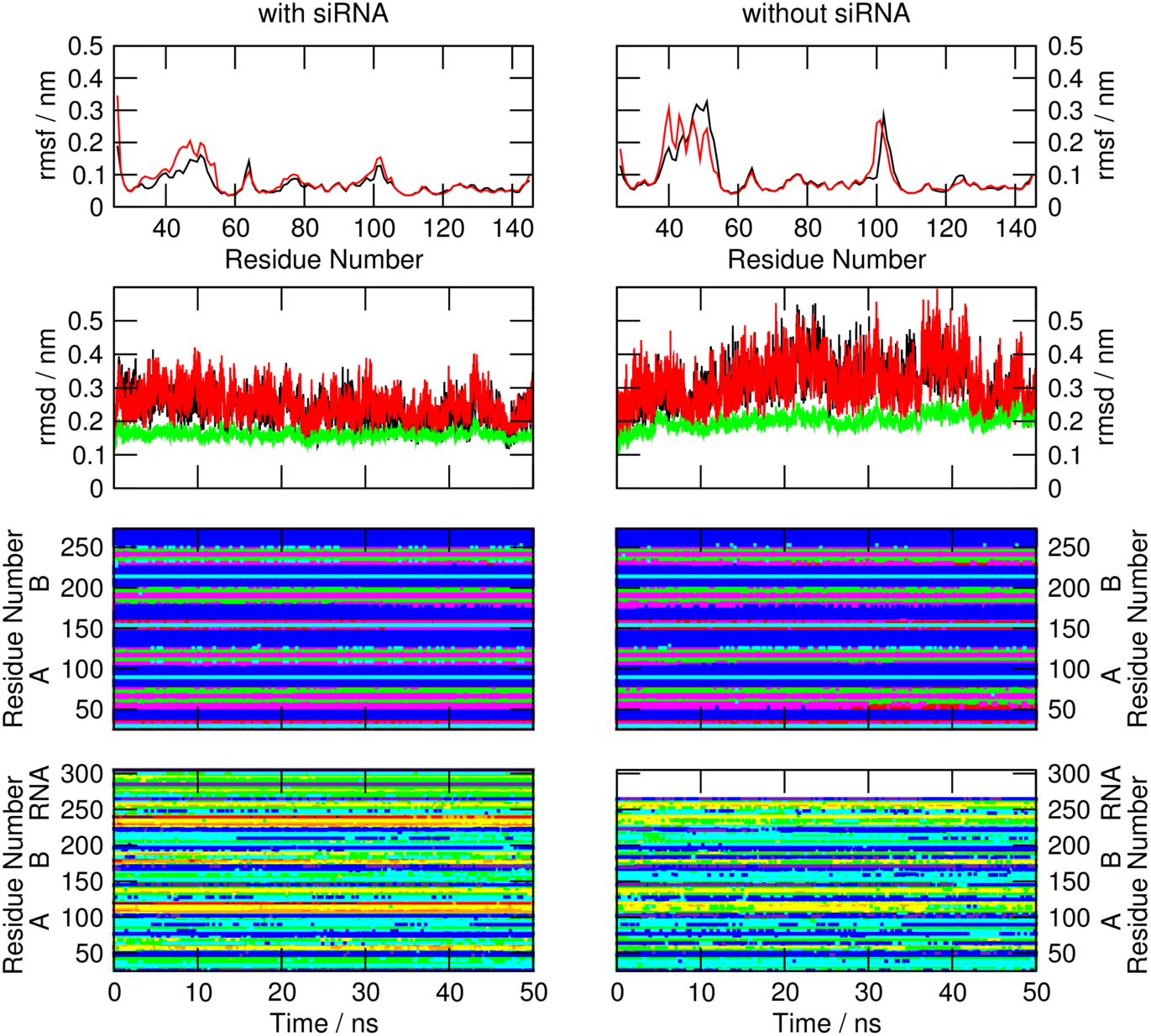
**

**Figure S:** Structural stability of the *permissible* p19 sequence variant R139W with and without a 19 bp siRNA bound**.**

**
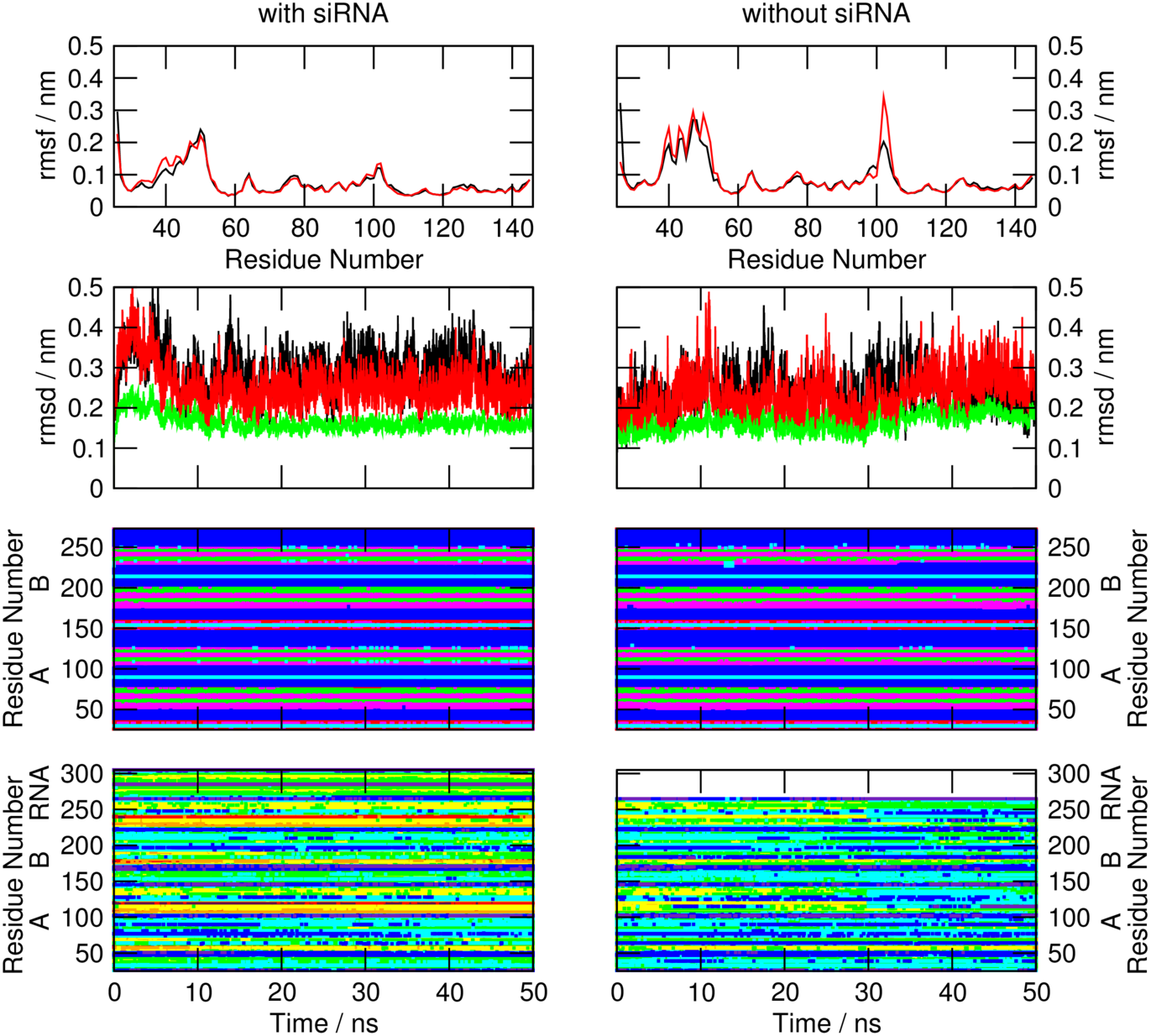
**

**Figure T:** Structural stability of the *permissible* p19 sequence variant G143A with and without a 19 bp siRNA bound**.**

**
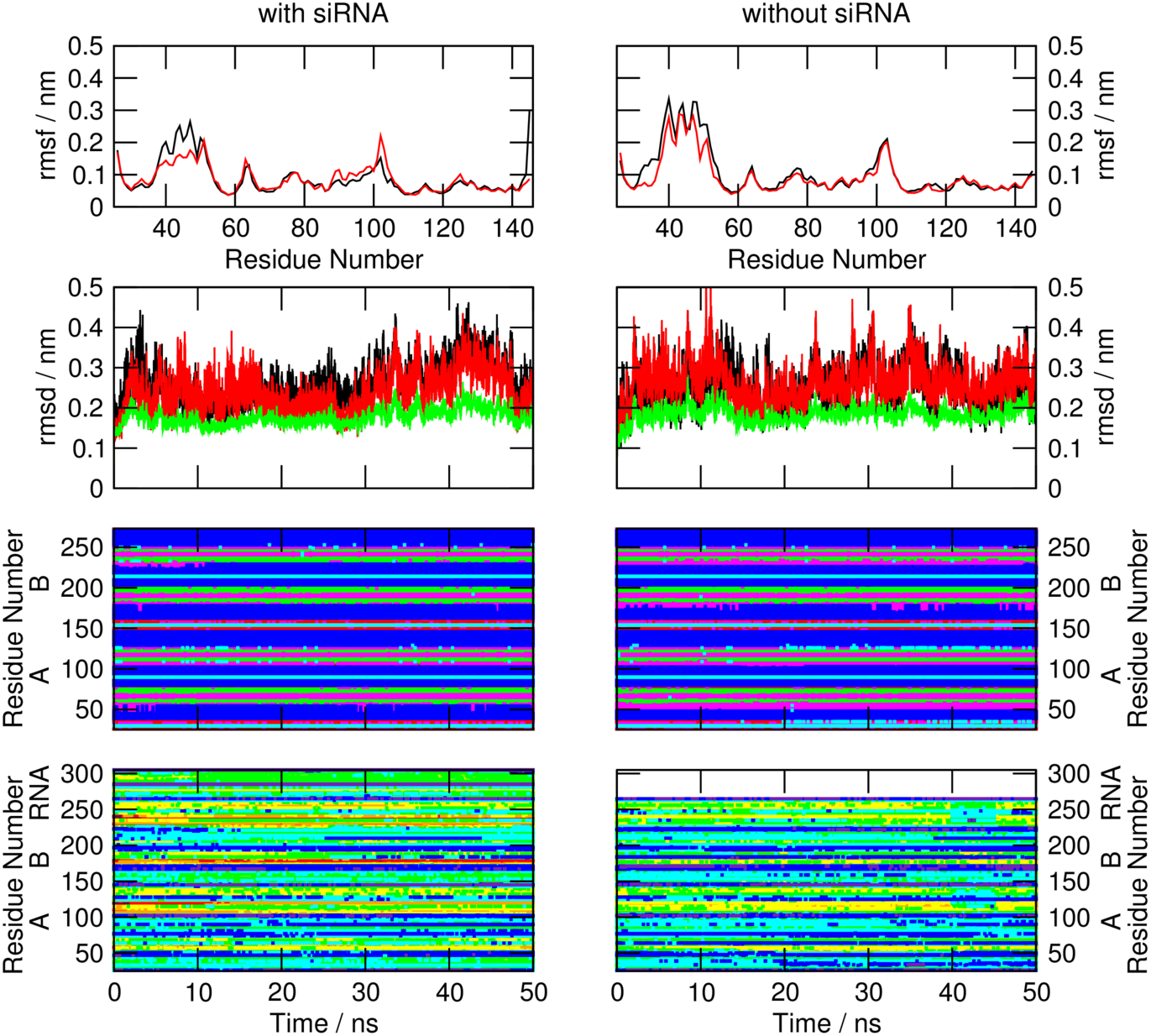
**

**Figure U:** Structural stability of the *permissible* p19 sequence variant G143S with and without a 19 bp siRNA bound**.**

**
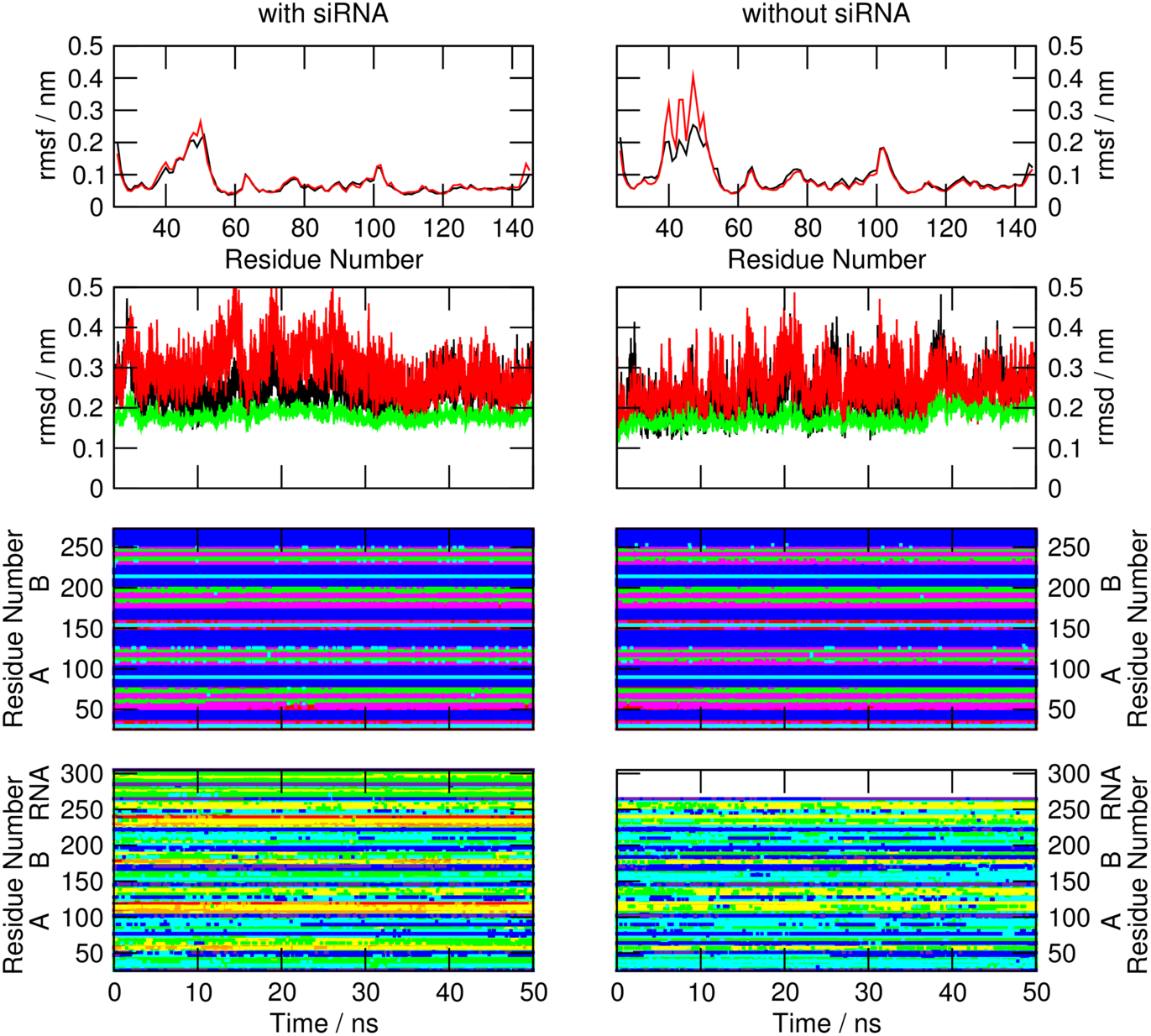
**

**Figure V:** Structural stability of the *permissible* p19 sequence variant G143T with and without a 19 bp siRNA bound**.**

**
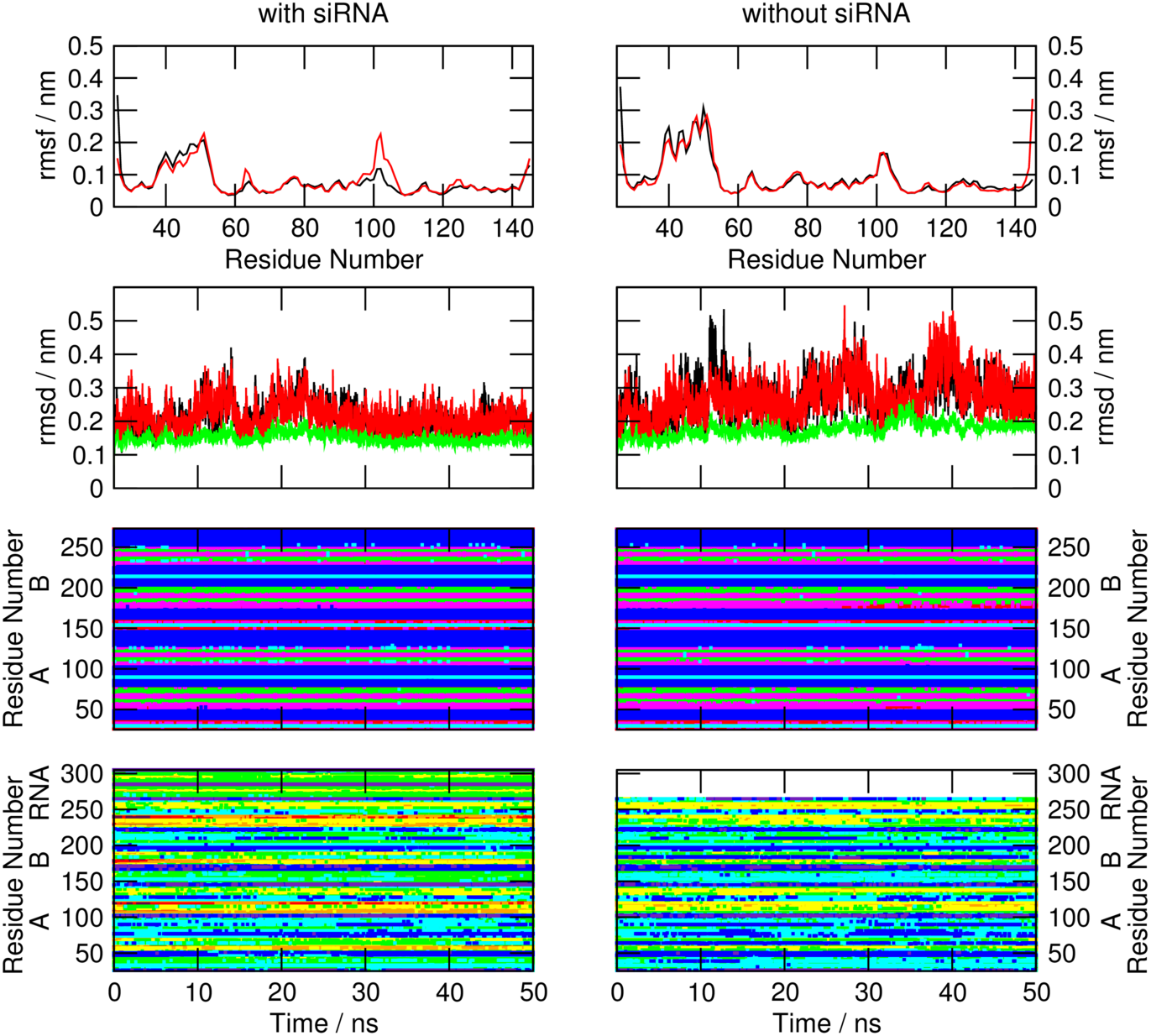
**

**Figure W:** Structural stability of the *permissible* p19 sequence variant G143Q with and without a 19 bp siRNA bound**.**

**
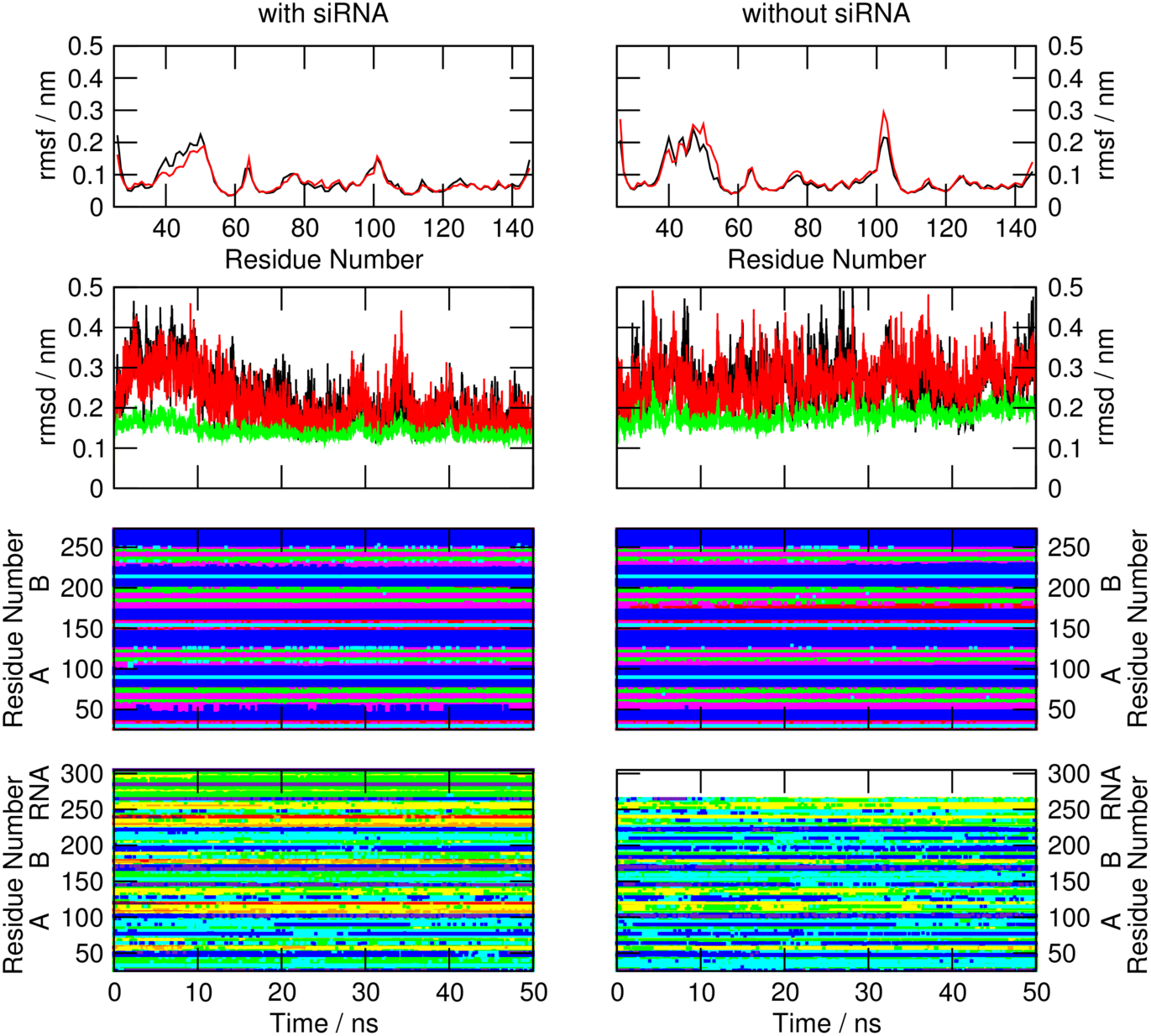
**

**Figure X:** Structural stability of the *permissible* p19 sequence variant G143K with and without a 19 bp siRNA bound**.**

**
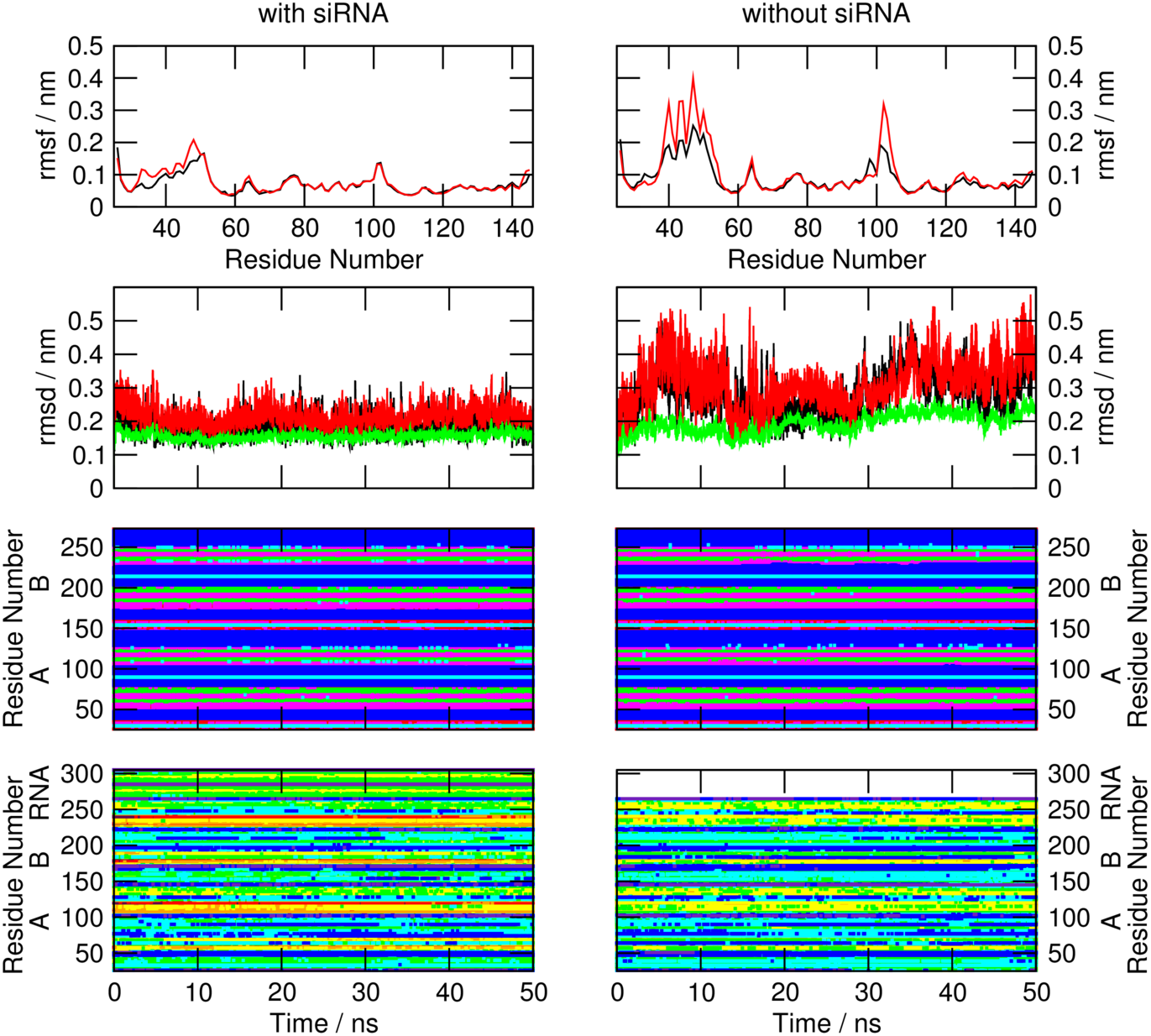
**

**Figure Y:** Structural stability of the *permissible* p19 sequence variant G143P with and without a 19 bp siRNA bound**.**

**
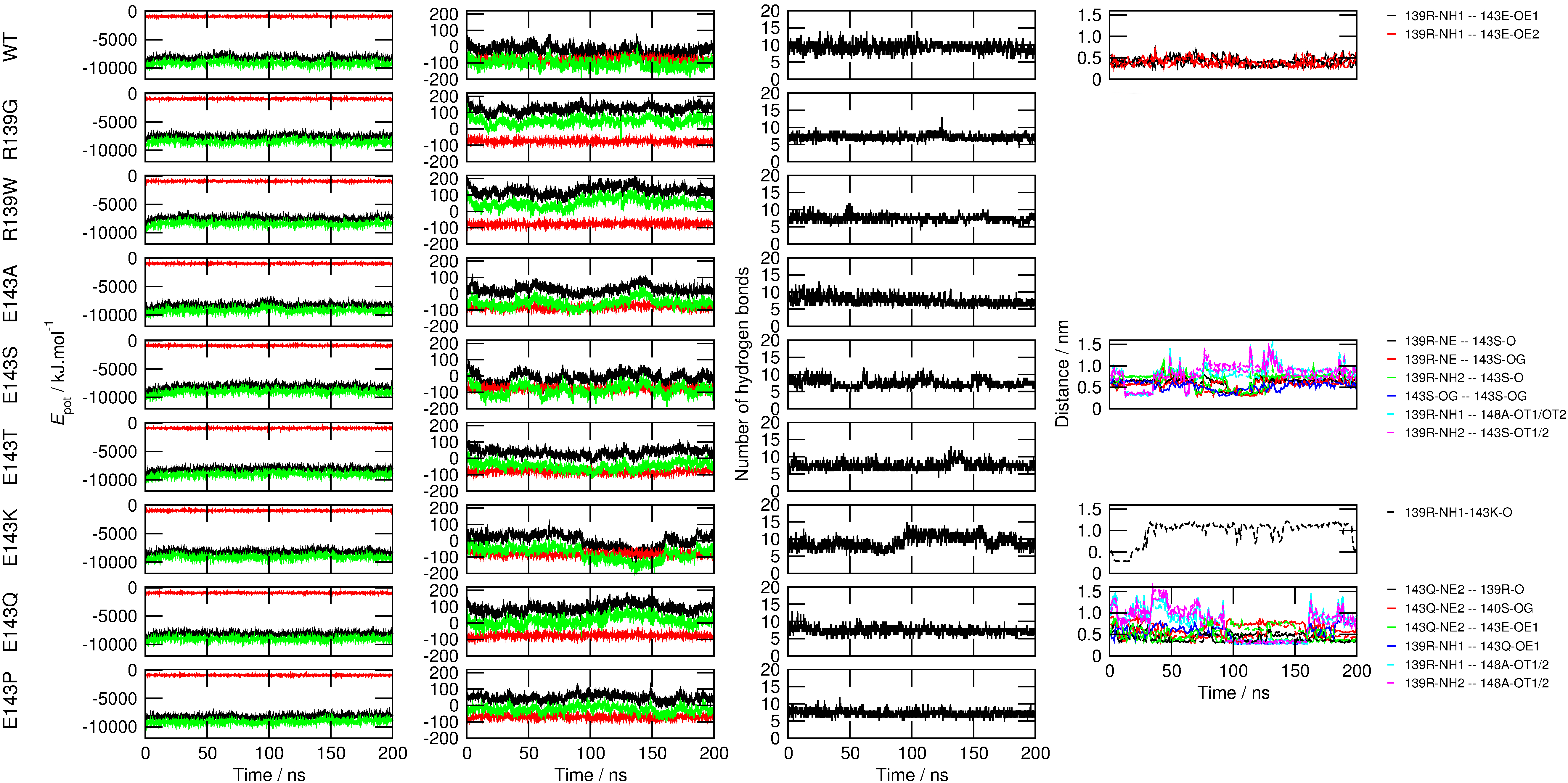
**

**Figure Z: Potential energies and dimer interface stability of all *permissible* variants of the wild-type tomato bushy stunt virus p19 sequence with a 19 bp siRNA bound during 200 ns molecular dynamics simulation.** The columns, from left to right, show the total potential energy of the entire protein/RNA/water system (black: Coulombic, red: van der Waals, green: total); the protein-protein interaction energy (same colouring); the total number of hydrogen bonds between the two protein subunits; the distance between the donor and acceptor atoms of residues forming hydrogen bonds or salt bridges across the dimer interface listed in Table K as labelled in the table legend. Interactions from the A to B subunits are shown as solid lines and those between identical atoms but from the B to A subunits are shown in dashed lines of the same colour.
